# Child and Parent Perceived Determinants of Children’s Inadequate Sleep Health. A Concept Mapping Study

**DOI:** 10.3390/ijerph17051583

**Published:** 2020-02-29

**Authors:** Laura S. Belmon, Vincent Busch, Maartje M. van Stralen, Dominique P.M. Stijnman, Lisan M. Hidding, Irene A. Harmsen, Mai J.M. Chinapaw

**Affiliations:** 1 Amsterdam UMC, Vrije Universiteit Amsterdam, Department of Public and Occupational Health, Amsterdam Public Health Research Institute, 1081 BT Amsterdam, The Netherlands; 2 Sarphati Amsterdam, Public Health Service (GGD), 1018 WT Amsterdam, The Netherlands; 3 Department of Epidemiology and Health Promotion, Section Youth, Municipal Health Service Amsterdam, 1018 WT Amsterdam, The Netherlands; 4 Department of Health Sciences, Faculty of Science and Amsterdam Public Health Research Institute, Vrije Universiteit Amsterdam, 1081 HV Amsterdam, The Netherlands

**Keywords:** sleep, childhood, children, determinants, factors, concept mapping

## Abstract

Many children do not meet the recommendations for healthy sleep, which is concerning given the potential negative effects on children’s health. To promote healthy sleep, it is crucial to understand its determinants. This concept mapping study therefore explores perspectives of children and parents on potential determinants of children’s inadequate sleep. The focus lies on 9–12 year old children (*n* = 45), and their parents (*n* = 33), from low socioeconomic neighbourhoods, as these children run a higher risk of living in a sleep-disturbing environment (e.g., worries, noise). All participants generated potential reasons (i.e., ideas) for children’s inadequate sleep. Next, participants sorted all ideas by relatedness and rated their importance. Subsequently, multidimensional scaling and hierarchical cluster analyses were performed to create clusters of ideas for children and parents separately. Children and parents both identified psychological (i.e., fear, affective state, stressful situation), social environmental (i.e., sleep schedule, family sleep habits), behavioural (i.e., screen behaviour, physical activity, diet), physical environmental (i.e., sleep environment such as temperature, noise, light), and physiological (i.e., physical well-being) determinants. These insights may be valuable for the development of future healthy sleep interventions.

## 1. Introduction

For children, healthy sleep is typically defined as a regular sleep rhythm consisting of approximately 9–11 hours per night [1] of good quality sleep (i.e., the combination of high sleep efficiency and a good subjective assessment of their own sleep) [2]. This nightly dose of healthy sleep is important for children’s cognitive performance [3,4], academic performance [3,5,6,7], and their physical and mental health [5,8,9,10]. Unfortunately, children’s average sleep duration has declined significantly in recent decades [11], while problems such as daytime sleepiness and longer sleep onset latency have become more prevalent [12,13]. This is especially apparent among children from lower socioeconomic (low SEP) neighbourhoods [14]. This may be ascribed to various environmental factors, such as noise nuisance caused by cramped housing or more parental stress caused by financial problems.

Knowledge of the most relevant determinants is essential for the development of effective interventions [15]. A recent review of prospective studies [16] found evidence that spending more time on screens (e.g., TV, computer, games), having a difficult temperament, and past poor or inadequate sleep health (i.e., sleep quality or quantity) were longitudinally associated with shorter sleep duration. Another review summarized empirical evidence related to common paediatric sleep recommendations and identified having an inappropriate bedtime, not having a relaxing bedtime routine, having an irregular sleep schedule, a negative emotional environment (e.g., family stress, family conflict), and poorer emotional well-being (e.g., higher levels of internalizing symptoms) as potential determinants of children’s inadequate sleep [17]. However, the perspectives of children and their parents are lacking in the current literature. 

The perspectives of children and their parents could bring about new and important insights into potential determinants of inadequate sleep, which can subsequently inform intervention development. Consequently, the aim of this study is to explore the perspectives of children and parents living in low-SEP neighbourhoods on potential determinants of children’s inadequate sleep health. 

## 2. Materials and Methods 

**Figure 1:**
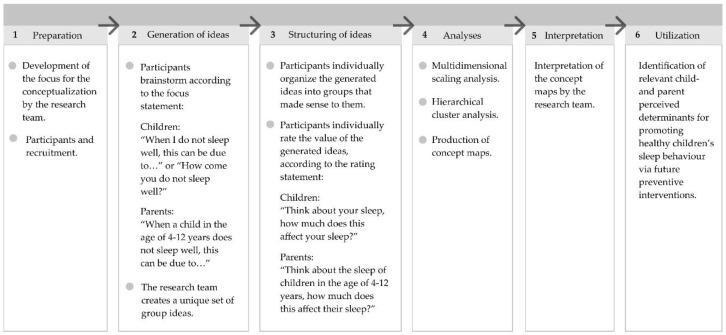
The 6-step concept mapping process.

A participatory mixed-methods concept mapping study was conducted to assess children’s and parents’ perspectives on potential determinants of children’s inadequate sleep health [18]. For the qualitative part of this approach, participants generated ideas about potential determinants during group brainstorm sessions, and subsequently rated these ideas according to importance. Researchers were not allowed to add or prompt additional ideas. Concept mapping is a 6-step process (see Figure 1). The first five steps are illustrated below. Step six includes the identification of perceived determinants that might be included in future healthy sleep interventions (see Discussion). Concept mapping has been used in previous studies into children’s perspectives on behavioural determinants [19,20]. Additional information about the concept mapping approach can be found elsewhere [18].

### 2.1. Preparation (Step 1)

The preparation phase included providing focus for the conceptualization, followed by identifying and recruiting participants. 

#### 2.1.1. Providing Focus for the Conceptualization

The first step was the creation of a ‘*focus statement*’ and ‘*rating statement*’: a main question or statement that gives a specific instruction for the session [18]. The purpose of the focus statement is to elicit ideas about the topic of interest, whereas the rating statement provides comparative ratings of importance for the generated ideas (see Figure 1). 

The comprehensiveness of the statements and the feasibility of the sorting and rating task were tested in a pilot study. For this, two pilot sessions were conducted with parents (9 and 6 parents, respectively) and two with children (12 and 26 children, respectively), and changes were made to clarify the focus- and rating statements. 

#### 2.1.2. Participants and Recruitment 

Children and parents were recruited through schools and thereby grouped based on school level and availability. The health advisors of the Public Health Service of Amsterdam brought the researchers in contact with primary schools in socioeconomically disadvantaged neighbourhoods, based on the postal code of the neighbourhood [21]. Four out of 23 invited primary schools participated. When schools were willing to participate, they were asked to distribute information letters to children aged 9–12 years old and parents with at least one child in the age range 4–12 years. The age range of the children (i.e., 9–12 years) was chosen because the tasks within the study were considered conceptually too difficult for children younger than 9 years old [22]. Participating children and at least one parent/caregiver provided informed consent. Schools were offered a lecture about healthy sleep as an incentive for participation, while children received a small present and parents a gift card with a value of 10 euros. The VU University Medical Ethical Committee approved the study protocol and concluded that it does not fall within the scope of the Medical Research Involving Human Subjects Act (study protocol 2017.013).

### 2.2. Generation of Ideas (Step 2)

At each primary school, two concept mapping sessions (1 to 1.5 hours) were organized per subgroup of children or parents, with approximately one week between sessions. The sessions were facilitated by one researcher (L.S.B.) and assisted by a second researcher (M.M.v.S, V.B., I.A.H., E.M.R., E.E.V., A.W., R.P. or L.B.). Each first session consisted of generating ideas in a brainstorm session. In each session, participants were first given a ‘warm-up question’ to stimulate understanding of the concept: “*What is inadequate sleep for you / for children?*”. Following this, they were encouraged to brainstorm individually about the *focus statement* (see Figure 1) and write down as many ideas as possible. Subsequently, everyone shared their ideas, one by one, with the rest of the group until none were left unmentioned, resulting in a complete list of original ideas per subgroup. During the first session, participants also completed a short questionnaire asking them about age, gender, education level (parents), and perceived cultural group (parents) or country of birth (children). Parents’ education categories were defined as 1) low, i.e., highest education level is primary or secondary school education or no education at all; 2) medium, i.e., highest education is secondary vocational education; and 3) high, i.e., highest education is higher professional education or scientific education. After each brainstorm session, ideas that were conceptually the same were merged, resulting in a set of unique ideas that were printed on cards for the second session. One researcher (L.S.B.) suggested the adaptations, which were checked by a second researcher (I.A.H.). In case of disagreement, a third researcher (V.B. or M.M.v.S.) was consulted. 

### 2.3. Structuring Ideas (Step 3) 

The second session consisted of structuring ideas. Firstly, participants individually sorted (i.e., clustered) all ideas by relatedness. The rules for this task were; 1) create a minimum of three and a maximum of 10 piles; 2) all cards (i.e., ideas) need to be placed on a pile; 3) a pile cannot consist of a single card (i.e., one idea); and 4) there cannot be a miscellaneous pile [18,19]. The last step of the sorting task was to name their piles of ideas. The name of each pile had to represent the relatedness between ideas. Secondly, they individually rated all ideas according to the rating statement: “*Think about your sleep, how much does this affect your sleep? / Think about the sleep of a child in the age of 4–12 years, how much does this affect their sleep?”* on a Likert scale: (1) ‘does not affect at all’ represented by a rested emoticon; (2) hardly affects; (3) affects a little; (4) affects a lot; (5) ‘affects a whole lot’ represented by a very tired emoticon [18]. 

### 2.4. Analyses (Step 4)

The software programme Ariadne [23] was used for the data analyses; multidimensional scaling, and hierarchical cluster analyses per subgroup [18]. We created a ‘two-dimensional point map’ on which each point represents an individual idea, with a specific ‘distance’ to the other ideas. Points that lie close to each other on the map represent ideas that were grouped together more often by participants. The table of similarities (or similarity matrix) structures the information of each participant about their perception of the relationship between ideas (i.e., the sorting task). 

### 2.5. Interpretation (Step 5)

**Figure A1:**
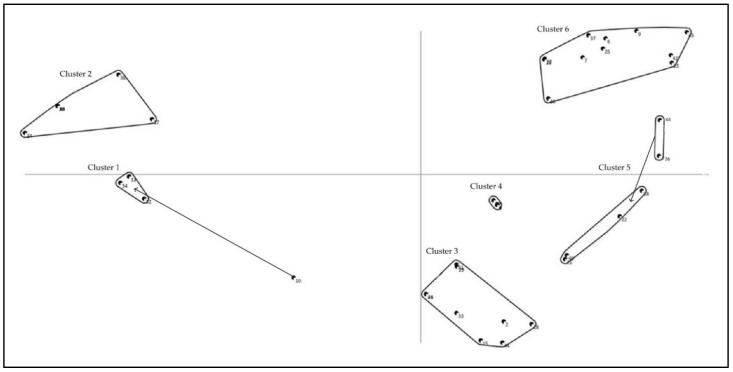
Concept map children, group 1. In this map, each point reflects one idea. Ideas that were grouped together more often appear closer to each other on the map. Ideas never/rarely grouped together appear widely separated on the map. Clusters are groups of ideas that were grouped together most often and reflect ideas that are conceptually related according to the participants in this group. The defined cluster names in this concept map were: Cluster 1: Screen behaviour; Cluster 2: Noise, distractions and light; Cluster 3: Being unable or reluctant to sleep; Cluster 4: Illness or restless; Cluster 5: Stressful and exciting thoughts and feelings; Cluster 6: Being afraid or uncomfortable in bed. Arrows indicate an idea is reallocated by researchers; idea 10 ‘Watching series in bed and wanting to keep watching’ was reallocated to Cluster 1, and idea 36 ‘Being bullied at school and thinking about the next day when I am in bed’ and 44 ‘Feeling sad’ were reallocated to Cluster 5.

**Figure A2:**
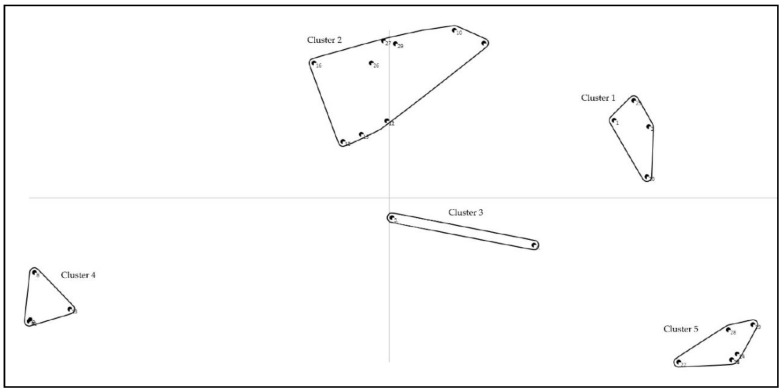
Concept map children, group 2. In this map, each point reflects one idea. Ideas grouped together more often appear closer to each other on the map. Ideas never/rarely grouped together appear widely separated on the map. Clusters are groups of ideas that were grouped together most often and reflect ideas that are conceptually related according to the participants in this group. The defined cluster names in this concept map were: Cluster 1: Being scared; Cluster 2: Stressful and exciting thoughts and feelings, and illness; Cluster 3: Light in the bedroom; Cluster 4: Noise and distractions in the bedroom; Cluster 5: Fear.

**Figure A3:**
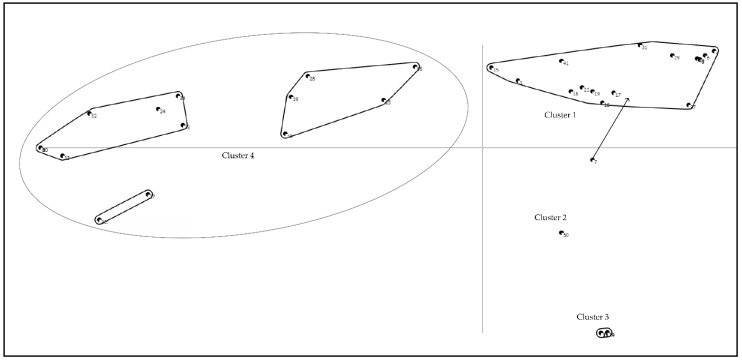
Concept map children, group 3A. In this map, each point reflects one idea. Ideas grouped together more often appear closer to each other on the map. Ideas never/rarely grouped together appear widely separated on the map. A circle means that a new cluster is created by the researchers, by combining original clusters. Clusters are groups of ideas that were grouped together most often and reflect ideas that were related conceptually according to the participants in this group. The defined cluster names in this concept map were: Cluster 1: Unable or reluctant to sleep; Cluster 2: Uncomfortable in bed; Cluster 3: Illness and discomfort; Cluster 4: Stressful and exciting thoughts and feelings. Arrows indicate an idea is reallocated by researchers; idea 7 ‘My parents who make noise in our home’ was moved to Cluster 1.

**Figure A4:**
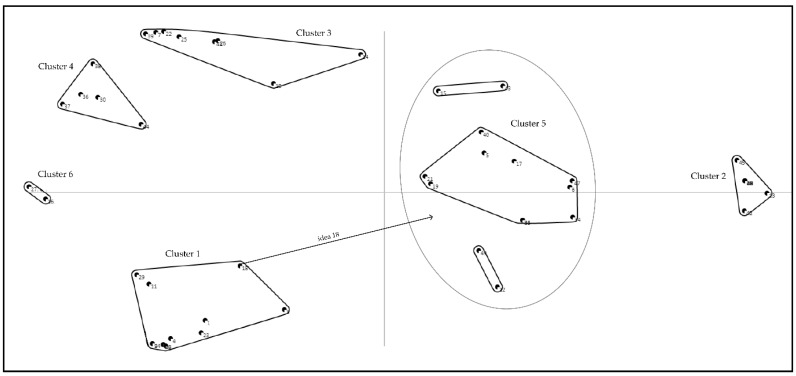
Concept map children, group 3B. In this map, each point reflects one idea. Ideas grouped together more often appear closer to each other on the map. Ideas never/rarely grouped together appear widely separated on the map. A circle means that a new cluster is created by the researchers, by combining original clusters. Clusters are groups of ideas that were grouped together most often and reflect ideas that were related conceptually according to the participants in this group. The defined cluster names in this concept map were: Cluster 1: Stressful and exciting thoughts and feelings; Cluster 2: Noise and distractions in the bedroom; Cluster 3: Illness and discomfort; Cluster 4: Emotions; Cluster 5: Unable or reluctant to sleep; Cluster 6: General negative state of mind. Arrows indicate an idea is reallocated by researchers; idea 18 ‘Not feeling like going to sleep’ was moved to Cluster 5.

**Figure A5:**
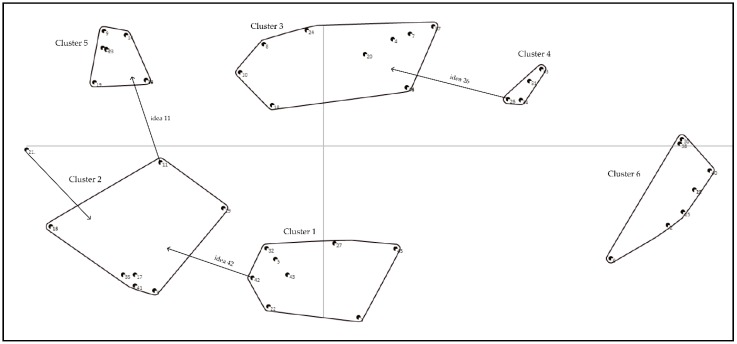
Concept map children, group 4A. In this map, each point reflects one idea. Ideas grouped together more often appear closer to each other on the map. Ideas never/rarely grouped together appear widely separated on the map. Clusters are groups of ideas that were grouped together most often and reflect ideas that were related conceptually according to the participants in this group. The defined cluster names in this concept map were: Cluster 1: Stressful thoughts and feelings; Cluster 2: Noise, distractions, and stressful family situation; Cluster 3: Unable or reluctant to sleep; Cluster 4: Stressed for the upcoming day; Cluster 5: Discomfort; Cluster 6: Being afraid. Arrows indicate an idea is reallocated by researchers; idea 11 ‘Needing to pee when I am already in bed’ was moved from Cluster 2 to Cluster 5; idea 21 ‘The sound of whizzing in my ears when it is very quiet’ was moved to Cluster 2; idea 26 ‘Still feel like playing’ was moved from Cluster 4 to Cluster 3; idea 42 ‘The sound of the TV is too loud when I am in bed’ was moved from Cluster 1 to Cluster 2.

**Figure A6:**
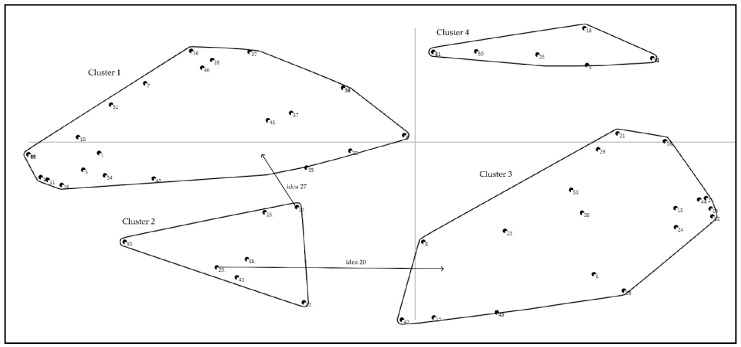
Concept map children, group 4B. In this map, each point reflects one idea. Ideas grouped together more often appear closer to each other on the map. Ideas never/rarely grouped together appear widely separated on the map. Clusters are groups of ideas that were grouped together most often and reflect ideas that were conceptually related according to the participants in this group. The defined cluster names in this concept map were: Cluster 1: Unable or reluctant to sleep; Cluster 2: Noise and distractions in the bedroom; Cluster 3: Stressful and exciting thoughts and feelings and stressful family situation; Cluster 4: Agitating activities before bedtime and being afraid. Arrows indicate an idea is reallocated by researchers; idea 20 ‘Sleeping at daytime’ was moved from Cluster 2 to Cluster 3; idea 27 ‘Thinking about someone I love and is not amongst us anymore’ was moved from Cluster 2 to Cluster 1.

**Figure A7:**
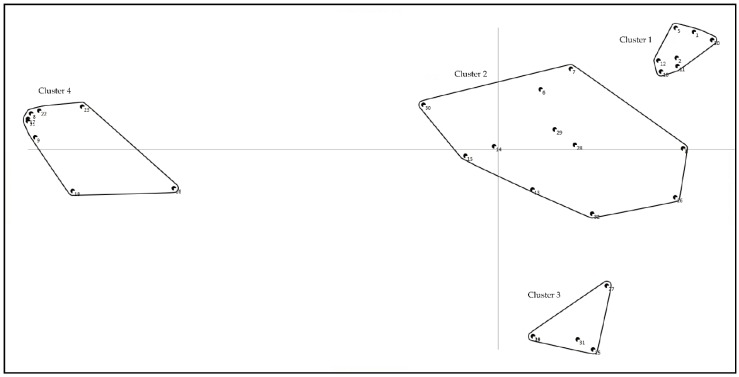
Concept map parents, group 1. In this map, each point reflects one idea. Ideas grouped together more often appear closer to each other on the map. Ideas never/rarely grouped together appear widely separated on the map. Clusters are groups of ideas that were grouped together most often and reflect ideas that were related conceptually according to the participants in this group. The defined cluster names in this concept map were: Cluster 1: Energy, diet, and physical activity; Cluster 2: Stressful and exciting thoughts and feelings, physical well-being, routines and screen behaviour; Cluster 3: Stressful family or school situation; Cluster 4: Sleep environment.

**Figure A8:**
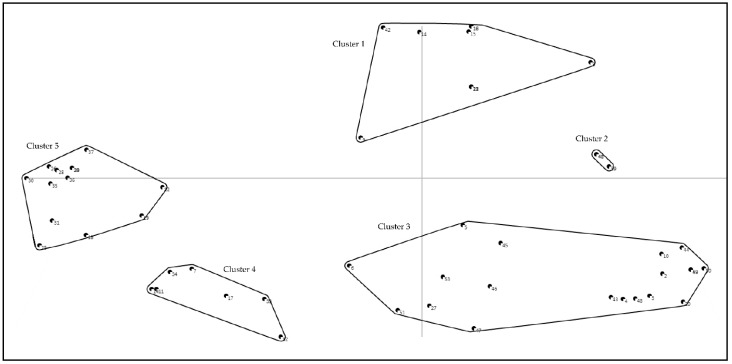
Concept map parents, group 2. In this map, each point reflects one idea. Ideas grouped together more often appear closer to each other on the map. Ideas never/rarely grouped together appear widely separated on the map. Clusters are groups of ideas that were grouped together most often and reflect ideas that were related conceptually according to the participants in this group. The defined cluster names in this concept map were: Cluster 1: Diet, physical activity, and screen behaviour; Cluster 2: Noise from the neighbours; Cluster 3: Mental distress, physical well-being, and stressful family situation; Cluster 4: Routines and social environment; Cluster 5: Sleep environment and bedtime routine.

**Figure A9:**
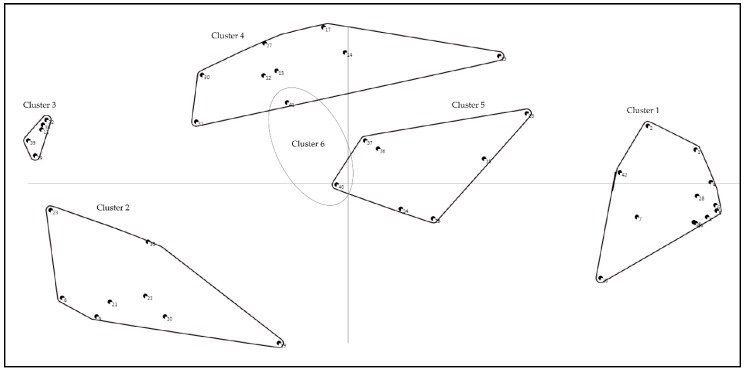
Concept map parents, group 3. In this map, each point reflects one idea. Ideas grouped together more often appear closer to each other on the map. Ideas never/rarely grouped together appear widely separated on the map. A circle means that a new cluster is created by the researchers, taking two ideas from two different clusters (cluster 4 and 5). Clusters are groups of ideas that were grouped together most often and reflect ideas that were related conceptually according to the participants in this group. The defined cluster names in this concept map were: Cluster 1: Concept map parents, group 3. Mental distress and active brain; Cluster 2: Family- and social environment; Cluster 3: Sleep environment; Cluster 4: Stressful family situation, worries and fear; Cluster 5: physical- and mental well-being; Cluster 6: Screen behaviour before bedtime.

**Figure A10:**
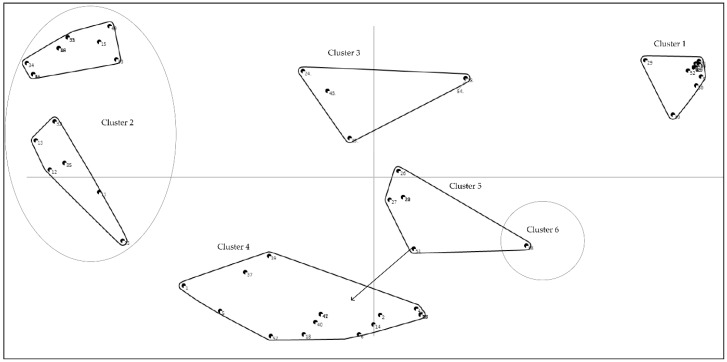
Concept map parents, group 4. In this map, each point reflects one idea. Ideas grouped together more often appear closer to each other on the map. Ideas never/rarely grouped together appear widely separated on the map. A circle means that a new cluster is created by the researchers; Cluster 2 is a combination of two identified clusters. Clusters are groups of ideas that were grouped together most often and reflect ideas that were related conceptually according to the participants in this group. The defined cluster names in this concept map were: Cluster 1: Sleep environment; Cluster 2: Mental distress, stressful family situation, worries, and fear; Cluster 3: Family environment and sleep habits; Cluster 4: Energy, diet, physical activity, and routines; Cluster 5: Screen behaviour and bedtime routine; Cluster 6: Physical well-being. Arrows indicate an idea is reallocated by researchers; idea 51 ‘Having a jetlag’ was moved from Cluster 5 to Cluster 4.

Two researchers (L.S.B. and D.P.M.S.) independently determined the optimal number of clusters for each subgroup using the divisive method and ‘hierarchical cluster tree’. For this method, all ideas start off in a single cluster. Based on the individual clustering of participants, the programme subsequently suggests how the ideas can be optimally arranged into clusters when choosing two, three, four, or more clusters. The underlying ideas in each cluster were interpreted incrementally and critically reviewed until each idea was in a cluster with other ideas that reflected a similar concept [18]. Potential conflicts were resolved by a third researcher (V.B.). Some ideas were moved to another cluster or a new cluster was created when this made more sense conceptually. These decisions can be found in Appendix A (Figure A1, Figure A2, Figure A3, Figure A4, Figure A5, Figure A6, Figure A7, Figure A8, Figure A9 and Figure A10) and B (Table A1, Table A2, Table A3, Table A4, Table A5, Table A6, Table A7, Table A8, Table A9 and Table A10). This process continued until consensus was reached on the number and meaning of the clusters per subgroup. After this, the clusters were named based on participants’ input. One concept map was created per subgroup. For the final concept maps see Appendix A. 

Clusters that represented multiple topics (i.e., perceived determinants) were split up. The original ideas were merged into main ideas for children and parents separately (see Appendix B). The average importance rating of each perceived determinant was calculated based on the average importance ratings of the underlying main ideas, and the average importance rating of each main idea was based on the average importance ratings of the underlying original ideas. An average ‘overall importance rating’ for all perceived determinants and main ideas was calculated by combining the mean rating of all participants across all groups, for children and parents separately. This created a clear overview of the importance of the perceived determinants and main ideas across all subgroups. An average rating of ≥3.00 was considered as important. Ratings between 2.95 and 2.99 were rounded to 2.9.

## 3. Results

### 3.1. Participants

The education level of the participating parents was mainly medium (42.4%) and high (42.4%). Six groups of children (*N* = 5 to 9) and four groups of parents (*N* = 7 to 10) were formed. Table 1 presents the sample characteristics.

### 3.2. Concept Maps

Children generated 30 to 58 ideas per group and parents 32 to 58 ideas. Some ideas generated in the first session were perceived as unclear or difficult in the subsequent session. In this case, the idea was excluded from sorting and rating (e.g., children in group 1 eventually discarded the idea ‘dancing in my bedroom at night’ and group 2 did so with ‘sleepwalking’). Two of the youngest children in group 1 experienced the second session as difficult and their clustering and ratings were therefore excluded from the analyses. The final number of clusters defined by the researchers ranged from four to six, for both children and parents. As the majority of these clusters represented multiple perceived determinants, we separated them to provide a clear overview of the different perceived determinants and their mean ratings. Table 2 presents children’s perceived determinants and the underlying ideas, categorized in five determinant domains: psychological, physiological, physical environmental, social environmental, and behavioural. Table 3 presents parents’ perceived determinants. In both tables, the determinants and underlying ideas are sorted from high to low importance.

### 3.3. Perceived Determinants of Children’s Inadequate Sleep

None of the child-perceived determinants were rated as important (≥3.00) by the children with an average score ranging from 1.9 to 2.9. However, two of the underlying main ideas were rated as important (≥3.00) and were mentioned by all groups of children: nightmares and illness. Other underlying main ideas that were mentioned by all groups of children but had a lower overall rating for importance were: noise from inside the house, distractions in the bedroom, excitement, negative affective state, and a recent stressful event. In addition, the underlying main ideas that did have a high importance score but were only mentioned in some groups of children were: a recent scary event, scary thoughts, not the right temperature, not being able to lie down comfortably, having too many thoughts, an upcoming stressful event, not being tired and going to bed too early. 

Almost all parent-perceived determinants and their underlying main ideas were rated as important (≥3.00) ranging from 2.9 to 3.9, and 2.1 to 4.4, respectively. The underlying main ideas that were mentioned by all groups of parents were: illness, parental relationship problems, being bullied, an upcoming stressful event, a recent stressful event, no consistent sleep schedule, watching something scary, screen use before bedtime, deviating from bedtime routine. The underlying main idea with the highest importance rating was feeling unsafe (mean rating of 4.4).

Most of the determinants mentioned by parents were also mentioned by children. However, the same ideas were often clustered differently by parents and children: 1) ‘physical well-being’ (parents) versus ‘discomfort’ and ‘diet’ (children); 2) ‘affective state’ and ‘stressful situation’ (parents) versus ‘affective state’ (children); 3) ‘energy’ and ‘physical activity’ (parents) versus energy (children); 4) ’sleep schedule’; 5) ‘sleep environment’; and 6) ‘activating activities’ (parents) versus ‘screen behaviour’ (children). Potential determinants that were mentioned by parents but not by children were ‘family sleep habits’ and ‘social environment’.

## 4. Discussion

The aim of this study was to explore potential determinants of children’s inadequate sleep health, from the perspective of school-aged children and parents living in low-SEP neighbourhoods. These perspectives brought about important insights, which can inform future intervention development to promote healthy sleep. Both children and parents identified various potential determinants of children’s inadequate sleep health which were categorized into psychological (i.e., fear, affective state), social environmental (e.g., sleep schedule, stressful situation), behavioural (e.g., screen behaviour, physical activity, diet), physical environmental (i.e., sleep environment), and physiological (i.e., discomfort, physical well-being) determinants. 

Fear was rated by children as most important. They described fear due to a recent scary event, reading or watching something scary, hearing scary sounds, or having a nightmare. Parents also rated affective state, including being afraid, as important. A recent review (2019) [16] found inconclusive evidence for a relationship between anxiety symptoms and sleep duration, and no evidence for a relationship with sleep quality. However, the results in this review are based on only two longitudinal studies. Additionally, Bagley et al. (2015) found that pre-sleep worries mediated the relationship between family income and children’s sleep health based on cross-sectional data [24]. Potentially, those who experienced anxiety engaged in unhelpful pre-bedtime behaviours [25]. Both children and parents in the current study rated the perceived psychological determinants, fear and affective state, as important, and these determinants included many underlying ideas. Consequently, dealing with fear or other negative affective feelings before falling asleep, concurrent with good sleep hygiene practices, may be a promising focus when promoting healthy sleep. This may be included in healthy sleep interventions by teaching children relaxation techniques, such as meditation, breathing exercises, imagination journeys [26] in both school, after-school and community settings. Additionally, teaching parents (i.e., online or through community programmes) how to implement such techniques in their child’s bedtime routine could be an important avenue for promoting healthy sleep.

Both children and parents identified a stressful situation (e.g., being bullied, parental stress, and parental relationship problems) as a potential determinant of inadequate sleep. This aligns with the results of a review that concluded that children who live in a supportive family environment and have a healthy relationship with their parents generally sleep longer and more efficiently [27]. A supportive and healthy family environment is characterized by parents’ involvement in their child’s life, and a good relationship between the child and its caregivers [27]. This also means parents should be aware of whether their child is going through a difficult time and support their child when needed (e.g., when the child has had a negative experience such as being bullied). Furthermore, one longitudinal study found that parent-child physical conflict (i.e., verbal aggression such as screaming, and physical aggression such as beating) was a determinant of children’s insufficient sleep duration [28]. Our findings support existing evidence that creating a positive and supportive family environment is important for healthy sleep. It may therefore be valuable to encourage parents to monitor their child’s needs, desires and stressors. Such interventions may include letting parents re-evaluate their behaviour, giving scenario-based risk information, or raising consciousness to increase parents’ awareness [15]. Increasing awareness must be quickly followed by increasing parent’s problem-solving ability and self-efficacy (i.e., confidence in their ability) by using methods such as goal setting, self-monitoring of behaviour, setting graded tasks, and planning coping responses [15]. This may reach parents via face-to-face or web-based sessions.

An inadequate sleep schedule, including an inappropriate bedtime (i.e., too early or late), an inconsistent sleep schedule (i.e., varying bedtimes throughout the week and weekend) and daytime napping, was identified by both children and parents as determinant of inadequate sleep. Additionally, parents identified the determinant ‘family sleep habits’, including not having or deviating from a bedtime routine, indistinctness about the child’s bedtime, and parental absence when the child needs attention. This finding aligns with the conclusions of previous reviews, and confirms parents’ critical role in children’s sleep hygiene [16,17,29]. Consequently, future sleep interventions may include enhancing parent’s self-efficacy and sleep-related parenting skills, e.g., to create and adhere to a consistent sleep schedule and a relaxing bedtime routine. Parents’ self-efficacy may be increased by using a method such as self-monitoring of behaviour, for which parents log the sleep schedule and bedtime routine activities of their child and subsequently receive feedback on these logs from a health professional [15].

Four perceived behavioural determinants were identified; energy, physical activity, activating activities, and dietary behaviour. It is generally accepted that adequate physical activity during the day promotes sleep at night [30,31]. This presumable relationship seems to be bidirectional, as the quantity and quality of sleep also seems related to physical activity the following day [32,33], creating a vicious circle [31]. Sleep is also related to diet [32,34]. Although most studies investigate inadequate sleep as a determinant of unhealthy dietary behaviours, the evidence for the bi-directionality of this relationship is growing [35]. In addition, a recent review among healthy adults [36] found that sleep was only impaired after vigorous intensity exercise, ending ≤ 1 h before bedtime. Furthermore, stimulating activities, including screen behaviours, are also generally acknowledged as important determinants of children’s inadequate sleep [37]. Such activities increase alertness, through several mechanisms: sleep time displacement, psychological stimulation and light exposure [38]. Encouraging sensible screen behaviours before bedtime (e.g., no agitating screen-based activities, avoiding screens in the bedroom) seems a promising element of future sleep interventions, but must be combined with other sleep hygiene strategies, e.g., regular bedtimes and a bedtime routine [38]. To conclude, these interactions and bidirectional relationships show that healthy sleep and its determinants should be included in healthy lifestyle interventions for children. To date, these interventions mainly focused on promoting a healthy diet and adequate amounts of physical activity [39].

Shortcomings in children’s sleep environment, including too much light, not the right temperature, noise, uncomfortable sleeping place, uncomfortable sleep materials, and distractions in the bedroom, were identified as potential determinants. This finding is partly in line with Bagley et al. who found that temperature and noise were associated with more sleep-wake problems in 10–13 year olds, while the amount of light was not related to any of their sleep outcomes [24]. The review by Allen et al. also showed limited support for adjusting the bedroom light to as dark (i.e., only two studies) and as quiet (i.e., only two studies) as possible for better sleep [17]. In contrast, a recent review of the WHO found evidence for a relationship between ambient noise and inadequate sleep [40]. Furthermore, a previous study (2017) where 11–12 year old multi-ethnic adolescents identified sleep-disturbing household activities, found that disorganization in the home environment such as TV or noise disturbance, family members phone calling, or night-time home visitors, were related to disturbed sleep [41]. In conclusion, environmental factors can be sleep-disrupting for some children, depending on children’s individual preferences for the amount of light, room temperature, noise level, and other distractions.

### 4.1. Strengths and Limitations

A strength of this study is that it provides valuable and new information about potential determinants of children’s inadequate sleep as seen from the perspective of children and their parents. These perspectives are relevant for future healthy sleep interventions. Moreover, this is one of the first studies that specifically focuses on families living in low SEP neighbourhoods. The potential determinants identified in this study may also be relevant for children living in middle and high SEP areas, however, future research is needed to confirm this. The broad focus on all aspects of inadequate sleep (duration, quality) further strengthens this study. However, there were also some limitations. Firstly, the concept mapping method makes use of a focus statement, which forces the researcher to choose one side of the health behaviour (i.e., positive or negative). This study focused on inadequate sleep, which inevitably neglects specific facilitators of healthy sleep. Secondly, potential personal determinants of which participants are unaware, e.g., unhelpful beliefs, may be overlooked. This may be included in future studies by incorporating follow-up questions focused on beliefs in the brainstorm session. Thirdly, it was not always clear why the children grouped certain ideas together. To give an example: a child (group 4A) clustered ‘In bed, thinking about something scary that I experience’ together with ‘A brother or sister that keeps me awake’. We therefore recommend that future concept mapping studies incorporate time to ask children to explain their clusters. Lastly, we did not screen the participating children and parents on potential sleep problems or other medical conditions. There is a possibility that poor sleepers and parents whose children have trouble sleeping were more interested in participating in this study despite the emphasis on recruiting children and parents with both adequate and inadequate sleep health.

Overall, all determinants were rated lower on importance by children than parents. A possible explanation may be found in the use of a smiley face Likert scale, as children tend to choose happy smiley faces rather than unhappy and very unhappy faces [42]. For future studies, we therefore recommend using smiley scales with only positive responses, varying in degree of happiness [42]. Although children’s importance ratings were lower in general, they still provided insight into the relative importance of determinants according to children.

### 4.2. Implications for Research and Practice

Our study identified potential determinants of children’s inadequate sleep health, of which the majority have not been studied thoroughly. Future research is therefore needed to confirm whether these are actual determinants of children’s inadequate sleep. In studying determinants of sleep, conducting a longitudinal study is not always required, as many psychological and social environmental determinants have acute effects. A suitable method would be ecological momentary assessment, for which potential determinants (e.g., affective state) are measured before bedtime and linked to subsequent sleep [43]. 

This study provides a broad overview of the perspectives of children and parents on potential determinants of children’s inadequate sleep. Our findings suggest that children’s sleep is affected by multiple interrelated and interacting determinants within the personal (i.e., physical, psychological and behavioural), social- and physical environment, which is in line with the social ecological model [15] and the ‘determinants of health’ model of Dahlgren and Whitehead [44]. This implies that a multilevel and multifactorial intervention is recommended to promote healthy sleep [45]. 

The results of our concept mapping study show that children’s inadequate sleep is a complex problem, with many different and interacting determinants on several levels of the social ecological model [15]. Such complex problems demand a systems approach [46,47], where relevant determinants of children’s sleep health are targeted at multiple levels of the system. Furthermore, children and parents identified many potential determinants in the current study. However, the relevance of these determinants might differ per child. For the development of a relevant healthy sleep intervention, we therefore recommend working closely with children and parents. 

## 5. Conclusions

Both children and parents identified various potential psychological (i.e., fear, affective state, stressful situation), social environmental (i.e., sleep schedule, family sleep habits), behavioural (i.e., screen behaviour, physical activity, diet), physical environmental (i.e., sleep environment), and physiological (i.e., physical well-being) determinants of children’s inadequate sleep health. These findings indicate that children and parents perceive children’s sleep health to be influenced by multiple determinants at different levels, and that children’s sleep health requires promotion through systemic, multilevel and multifactorial action. 

## Figures and Tables

**Table 1:** Sample characteristics.

Characteristics		
**Children (*N* = 45)**		
	Age, years (M, SD)	10.2 (1.1)
	Female (%)	62.2
	Born in the Netherlands (%)	88.9
**Parents (*N* = 33)**		
	Age, years (M, SD)	40.4 (7.9)
	Female (%)	90.9
	Education level (%)	
	Low	9.1
	Medium	42.4
	High	42.4
	Unknown	6.1
	Perceived cultural group (%)	
	Dutch	36.4
	Turkish	15.2
	Moroccan	6.1
	Ghanaian	3.0
	Other	12.1
	Two or more groups	27.3

*N* = number of participants; M = Mean; SD = Standard deviation.

**Table 2:** Mean importance ratings ^1^ for the child-perceived determinants related to children’s inadequate sleep.

Perceived Determinants	Main Ideas (Merged)	Examples of Underlying Original Ideas	Mean Rating per Child Group ^2^						Mean ^3^
			*1*	*2*	*3A*	*3B*	*4A*	*4B*	
***Psychological determinants***									
**Fear**									*2.9*
	Recent scary event	*‘In bed, thinking about something scary that I experienced’*	N.A.	**3.6**	N.A.	N.A.	**3.1**	**3.3**	**3.3**
	Scary thoughts	*‘Having scary thoughts when I am in bed’*	**3.3**	**3.6**	2.9	N.A.	N.A.	N.A.	**3.3**
	Nightmares	*‘Having a nightmare’*	**3.1**	**3.2**	**3.2**	2.8	**3.0**	**3.2**	**3.1**
	Watch/read something scary	*’Having seen a scary movie’, ‘Reading a moving story before I go to sleep’*	2.7	**3.0**	N.A.	2.6	**3.1**	2.7	2.8
	Scared by something in the bedroom	*‘When I see scary shadows in my bedroom’*	1.4	2.2	**3.4**	N.A.	N.A.	**3.3**	2.6
	Being afraid	*‘Being afraid when I am in bed’*	2.4	N.A.	2.8	2.4	2.1	**3.2**	2.6
	Scary sounds	*‘Hearing weird or scary sounds during the night’*	N.A.	N.A.	2.6	2.4	N.A.	N.A.	2.5
**Affective state**									*2.6*
	Many thoughts	*‘Thinking and having many thoughts’*	N.A.	N.A.	**3.8**	**4.3**	N.A.	2.8	**3.6**
	Upcoming stressful event	*‘Being nervous for something that is going to happen’*	**3.3**	**3.0**	N.A.	2.9	**3.5**	2.6	**3.1**
	Excitement	*‘Looking forward to something that will happen the next day’*	2.6	**3.0**	**3.0**	**3.1**	**3.3**	2.8	2.9
	Negative affective state	*‘Feeling sad’, ‘Being angry’, ‘Being irritated’*	2.4	2.9	2.8	2.5	**3.0**	**3.3**	2.8
	Reluctant to go to sleep	*‘Not feeling like going to sleep’*	2.4	N.A.	2.7	2.4	**3.0**	**3.6**	2.8
	Recent stressful event	*’Continuing to think about a bothersome event that happened that day’*	2.3	2.5	**3.0**	2.5	2.2	2.9	2.6
	Stressful family situation	*‘A fight between my parents when I am in bed’*	N.A.	N.A.	N.A.	2.2	3.4	1.7	2.4
	Feeling unsafe	*‘Not feeling comfortable because of people screaming outside’*	N.A.	N.A.	N.A.	2.3	2.2	2.1	2.2
	Fear of missing out	*‘When my brother/sister is allowed to watch something (TV, film) and I am not’*	N.A.	N.A.	2.0	N.A.	N.A.	N.A.	2.0
	Lacks attention from parents	*‘When my parents do not pay attention to me because they are busy with my brother or sister’*	N.A.	N.A.	2.0	N.A.	N.A.	N.A.	2.0
*Physiological determinants*									
**Discomfort**									*2.8*
	Illness	*‘Being ill’, ‘Having a blocked nose due to a cold’*	**3.1**	**3.4**	2.9	**3.5**	**3.9**	**3.2**	**3.3**
	Pain	*‘Feeling pain’*	2.9	**3.0**	N.A.	2.5	**3.6**	2.2	2.8
	Needing to pee	*‘Needing to pee when I am already in bed’*	2.8	N.A.	N.A.	2.6	2.7	2.4	2.6
	Unhealthy dietary behaviour	*‘Having had too much to eat’, ‘Late dinner’, ‘Feeling hungry’*	N.A.	N.A.	N.A.	2.4	2.7	2.6	2.6
*Physical environmental determinants*									
**Sleep environment**									*2.7*
	Not the right temperature	*‘Feeling too hot or too cold when I am in bed’*	**3.8**	N.A.	**3.0**	2.8	**3.6**	2.9	**3.2**
	Unable to lie down comfortably	*‘Not able to lie down comfortably in my bed’*	**3.8**	N.A.	**3.2**	2.4	N.A.	**3.2**	**3.1**
	Noise outside	*‘Noise from the neighbours, e.g., yelling or music’*	**3.0**	**3.3**	N.A.	2.4	2.9	**3.1**	2.9
	Uncomfortable sleeping materials	*‘Having no comfortable pillow’, ‘Wearing uncomfortable pyjamas’*	2.9	N.A.	**3.2**	N.A.	N.A.	2.7	2.9
	Uncomfortable bed	*‘Sleeping on an uncomfortable mattress’*	N.A.	N.A.	N.A.	N.A.	N.A.	2.9	2.9
	Too much light	*‘Too much light in my bedroom’*	2.9	**3.0**	N.A.	2.4	N.A.	2.8	2.8
	Noise inside	*‘When the sound of the TV in our home is too loud’*	2.8	2.2	2.5	2.4	2.9	2.59	2.6
	Distractions in the bedroom	*‘Pets that wake me up’, ‘Noise from a brother or sister with whom I share the same room’*	2.4	2.4	2.1	2.0	2.5	2.2	2.3
	Unfavourable sleeping place	*‘Not sleeping at my favourite sleeping place in the bunk bed’*	2.1	N.A.	N.A.	N.A.	N.A.	N.A.	2.1
	Too dark	*‘A bedroom that is too dark’*	N.A.	1.8	N.A.	N.A.	N.A.	N.A.	1.8
*Social environmental determinants*									
**Sleep schedule**									*2.6*
	Going to bed too early	*‘Going to bed too early and not being tired enough to fall asleep’*	2.8	N.A.	**3.2**	**3.6**	**3.3**	N.A.	**3.2**
	No consistent sleep schedule	*‘Not going to bed at the same time every night’*	N.A.	N.A.	2.6	2.0	1.9	2.2	2.2
	Daytime nap	*‘Sleeping at daytime’*	N.A.	N.A.	N.A.	N.A.	2.7	2.0	2.4
*Behavioural determinants*									
**Energy**									*2.4*
	Not being tired	*‘Not being tired when I go to bed’*	**3.9**	N.A.	N.A.	N.A.	**3.1**	N.A.	**3.5**
	Inadequate amount of daytime PA	*‘Having had too little exercise during the day and therefore too much energy in the evening’*	2.1	N.A.	N.A.	2.5	N.A.	N.A.	2.3
	Excessive daytime stimulation	*‘Being too tired because I did a lot’*	N.A.	N.A.	N.A.	1.9	N.A.	2.22	2.1
	Evening PA	*‘Doing sports late in the evening’*	1.5	N.A.	2.4	N.A.	1.9	N.A.	1.9
**Screen behaviour**									1.9
	Screen use around bedtime	*‘Using a phone or tablet before bedtime’*	2.5	2.0	N.A.	2.1	1.9	1.9	2.1
	Social media use around bedtime	*‘Messages I receive in the evening* via *the group chat on my phone’*	1.7	2.0	N.A.	N.A.	N.A.	N.A.	1.9
	Playing activating games before bedtime	*‘Playing computer games right before going to sleep’*	1.4	N.A.	N.A.	2.0	N.A.	N.A.	1.7

N.A. = Not applicable, means the idea was not mentioned in this group of children; bold values indicate the determinant or idea was perceived as important i.e., ≥3.00; cursive values indicate the overall mean rating per perceived determinant. PA = Physical activity. ^1^ The mean importance rating is based on the question: ‘Think about your sleep, how much does this affect your sleep?’ answered on a 5-point Likert scale from ‘does not affect at all = 1’ to ‘affects a whole lot’ = 5’. ^2^ Groups of children per school (i.e., schools 1–4). At schools 3 and 4, there were two groups of children, represented by A and B. ^3^ Mean importance rating of all groups of children.

**Table 3:** Mean importance ratings ^1^ for the parent-perceived determinants related to children’s inadequate sleep.

Perceived Determinants	Main Ideas (Merged)	Examples of Underlying Original Ideas	Mean Rating per Parent Group ^2^				Mean ^3^
			*1*	*2*	*3*	*4*	
***Physiological determinants***							
**Physical well-being**							***3.9***
	Illness	*‘Being ill’*	**4.1**	**4.2**	**4.2**	**3.5**	**4.0**
	Pain	*‘Feeling pain’*	N.A.	N.A.	**4.2**	N.A.	**4.2**
	Sleep problem	*‘Sleep walking’, ‘Wetting the bed’*	**3.0**	N.A.	N.A.	**4.0**	**3.5**
*Psychological determinants*							
**Stressful situation**							***3.8***
	Feeling unsafe	*‘Not feeling safe at home, in the classroom or outside on the streets’*	N.A.	N.A.	**4.1**	**4.7**	**4.4**
	Parental relationship problems	*‘Negative tension or disagreement within the family’, ‘Parents going through a divorce’*	**3.4**	**3.8**	**4.5**	**4.4**	**4.0**
	Being bullied		**3.4**	**3.8**	**4.2**	**4.7**	**4.0**
	Insecurity about themselves	*‘Feeling insecure about themselves’*	**4.0**	N.A.	N.A.	N.A.	**4.0**
	Parental stress	*‘Parental stress (rushing) that is transferred to the child’*	**3.9**	N.A.	**3.1**	**4.3**	**3.8**
	Financial family problems	*‘Financial problems at home, meaning the parent is unable to buy everything for the child’*	N.A.	2.6	N.A.	N.A.	2.6
**Affective state**							***3.6***
	Unpleasant dreams	*‘Nightmares, ‘Dreams that keep children awake or wake them and cause restless sleep’*	N.A.	N.A.	**3.3**	**4.5**	**3.9**
	Upcoming stressful event	*‘A stressful event coming up for the child the next day’*	**4.0**	**3.8**	**3.6**	**3.5**	**3.7**
	Being afraid	*‘Being afraid when lying in bed’*	N.A.	**3.6**	3.1	**4.5**	**3.7**
	Many thoughts	*‘Having many thoughts’*	N.A.	N.A.	**3.3**	**4.2**	**3.7**
	Recent stressful event	*‘Continuing to think about something that happened that day’*	**3.9**	2.9	**3.6**	**4.3**	**3.7**
	Excitement	*‘Excitement, happy feelings for something that is going to happen the next day’*	**3.6**	**3.6**	N.A.	**3.2**	**3.4**
	Fear of missing out	*‘Not willing to miss something and therefore not willing to go to sleep’*	N.A.	**3.9**	**3.1**	**3.2**	**3.4**
	Worrying	*‘Worrying about something and not being able to share this’*	**3.6**	2.9	**3.7**	N.A.	**3.4**
	Reluctant to go to sleep	*‘Not feeling like going to sleep’*	**3.0**	N.A.	N.A.	**3.3**	**3.2**
*Behavioural determinants*							
**Energy**							***3.5***
	Being too energetic	*‘Not being tired when going to bed’, ‘Having too much energy from his/herself’*	**3.9**	N.A.	N.A.	**3.8**	**3.8**
	Excessive daytime stimulation	*‘A busy day with excessive stimulation due to too many activities’*	N.A.	N.A.	**3.4**	**4.0**	**3.7**
	Inadequate daytime stimulation	*‘A boring day with inadequate stimulation due to lack of activities’*	**3.6**	N.A.	**3.1**	**3.2**	**3.3**
	Being too tired	*‘Being too tired when going to bed’*	**3.3**	N.A.	**3.6**	2.8	**3.2**
**Activating activities**							**3.4**
	Watching something scary	*‘Watching a scary movie’, ‘Watching the news’*	**3.4**	**4.0**	**3.6**	**3.8**	**3.7**
	Play with activating toys before bedtime	*‘Playing with toys with a lot of light and noise right before bedtime’*	N.A.	**3.1**	**4.4**	N.A.	**3.8**
	Screen use before bedtime	*‘Using the computer or other screen (phone, tablet, game computer, TV) right before going to sleep’*	**3.4**	**3.3**	**3.7**	**3.3**	**3.4**
	Excessive daytime screen use	*‘Using screens (phone, tablet, game computer, TV) a lot during the day’*	N.A.	N.A.	N.A.	2.7	2.7
**Physical activity**							***3.3***
	Inadequate amount of daytime PA	*‘Inadequate amount of physical activity during the day and therefore not being tired’*	N.A.	**3.6**	N.A.	**3.2**	**3.4**
	Inadequate time outside at daytime	*‘Not having played outside at daytime’, ‘Spending too little time outside in the fresh air’*	**3.0**	N.A.	N.A.	**3.3**	**3.2**
**Diet**							***3.1***
	Unhealthy diet	*‘Eating something sugary before bedtime’, ‘Unhealthy diet during the day’*	**3.4**	**3.6**	N.A.	2.5	**3.2**
	Excessive amount of food close to bedtime	*‘Eating too much right before going to bed’*	**3.3**	**3.5**	N.A.	2.7	**3.2**
	Drinking too much before bedtime	*‘Drinking too much before going to sleep and therefore needing to go to the toilet often’*	N.A.	N.A.	N.A.	**3.2**	**3.2**
	Did not drink enough	*‘Feeling thirsty’, ‘Lack of water during the day’*	N.A.	N.A.	N.A.	**3.0**	**3.0**
	Inadequate amount of food	*‘Feeling hungry during the night’*	2.6	N.A.	N.A.	**3.2**	2.9
*Social environmental determinants*							
**Sleep schedule**							***3.5***
	Too early bedtime	*‘A bedtime that is too early for child’s circadian rhythm’*	N.A.	N.A.	N.A.	**3.8**	**3.8**
	No consistent sleep schedule	*‘No consistent sleep times’, ‘Irregular sleep times during weekends’*	**3.9**	**3.5**	**3.4**	2.8	**3.4**
	Daytime nap	*‘Napping in the afternoon’*	N.A.	**3.0**	N.A.	**3.5**	**3.3**
**Family sleep habits**							**3.3**
	No bedtime routine	*‘Having no bedtime routine’*	N.A.	**3.3**	N.A.	**3.7**	**3.5**
	Parental absence	*‘Absence of the mother or father when the child needs attention’*	N.A.	**4.0**	N.A.	2.9	**3.5**
	Indistinctness about bedtime	*‘Not indicating clearly when the child needs to go to bed’*	N.A.	**3.2**	N.A.	N.A.	**3.2**
	Deviate from bedtime routine	*‘When the parent deviates from the usual bedtime routine’*	2.6	2.7	**3.4**	**3.5**	**3.1**
**Social norms**							*2.9*
	Social bedtime norm among siblings	*‘Older brothers or sisters that are allowed to stay up longer’*	N.A.	**4.2**	2.7	2.8	**3.2**
	Social bedtime norm among classmates	*‘Other children in their class that are allowed to stay up longer’*	N.A.	2.8	2.3	N.A.	2.6
*Physical environmental determinants*							
**Sleep environment**							***3.0***
	Seasonal changes	*‘When it is still light outside when they need to go to bed’*	2.9	**4.0**	N.A.	**3.2**	**3.4**
	Absence of favourite sleep accessory	*‘The absence of their favourite stuffed animal or sleeping cloth’*	N.A.	2.7	**3.1**	**4.2**	**3.3**
	Noise outside	*‘Fighting neighbours’, ‘Noise from the street’*	N.A.	**3.6**	2.9	**3.5**	**3.3**
	Not the right temperature	*‘A bedroom that is too hot or too cold’*	**3.1**	**3.4**	N.A.	**3.1**	**3.2**
	Reluctant to sleep alone	*‘Wanting to stay with their parent and not wanting to be alone’*	**3.3**	N.A.	**3.2**	**3.0**	**3.2**
	Uncomfortable sleeping material	*‘Uncomfortable sleeping attributes, such as pillows, pyjamas, underwear’*	N.A.	**3.1**	N.A.	N.A.	**3.1**
	Too much light	*‘Too much light in the bedroom’*	2.7	**3.4**	N.A.	**3.0**	**3.1**
	Noise inside	*‘Too much noise within the home’*	N.A.	N.A.	**3.0**	**3.0**	**3.0**
	Different sleep environment	*‘A different environment, not sleeping in their own bed’*	N.A.	**3.0**	N.A.	**3.0**	**3.0**
	Uncomfortable place to sleep	*‘Not having a comfortable bed and therefore not being able to lay down comfortably’*	N.A.	**3.0**	N.A.	**3.0**	**3.0**
	No fresh air	*‘No fresh air in the bedroom’*	N.A.	**3.0**	N.A.	N.A.	**3.0**
	Distractions in the bedroom	*‘A less tidy and messy bedroom’, ‘Shared bedroom with brothers or sisters’*	2.8	**3.0**	N.A.	**3.2**	2.9
	Too dark	*‘A bedroom that is too dark’*	2.9	2.9	2.9	N.A.	2.9
	Too quiet	*‘When it is too quiet at home’*	N.A.	N.A.	2.1	N.A.	2.1

N.A. = Not applicable, means the idea was not mentioned in this group of parents; bold values indicate that the determinant or idea was perceived as important i.e., ≥3.00; cursive values indicate the overall mean rating per perceived determinant; PA = Physical activity. ^1^ The average importance ratings were based on the question: ‘Think about the sleep of a child in the age of 4–12 years, how much does this affect their sleep?’ answered on a 5-point Likert scale from ‘does not affect at all = 1’ to ‘affects a whole lot = 5’. ^2^ Groups of parents per school (i.e., schools 1–4). ^3^ Mean importance rating of all groups of parents.

## Abbreviations

A.W.: Anouk Wisse; D.P.M.S.: Dominique Patricia Maria Stijnman; E.M.R.: Eline Maria Roordink; E.E.V.: Eline Eva Vos; I.A.H.: Irene Anhai Harmsen; L.S.B.: Laura Shanna Belmon, L.B.: Liesbet Boterdaele; L.M.H.: Lisan Mariët Hidding; M.M.v.S.: Maartje Marieke van Stralen; M.J.M.C.: Mai Jeanette Maidy Chinapaw; R.P.: Rian Pepping; V.B.: Vincent Busch.

## Appendix B

**Table A1:** Cluster compositions of original ideas and mean importance ratings of children in group 1 (*N* = 8), including the assignment of original ideas to main ideas (*N* = 6).

Clusters	Original Ideas	Idea nr. ^1^	Merged to Main Idea ^2^	Mean ^3^
**1. Screen behaviour**				*2.1*
	Using a phone or tablet before bedtime	*1*	Screen use around bedtime	2.5
	Watching series in bed and wanting to keep watching ^4^	*10*	Screen use around bedtime	2.9
	Receiving messages on my phone from others when I am in bed	*11*	Social media use around bedtime	1.9
	Using Instagram, Snapchat or other social media when I am in bed	*12*	Social media use around bedtime	1.5
	Playing a game before bedtime	*33*	Playing activating games before bedtime	1.4
	Watching TV before bedtime	*35*	Screen use around bedtime	2.1
**2. Noise, distractions and light**				*2.9*
	Noise from cars outside	8	Noise outside	**3.1**
	Noise from the neighbours, e.g., yelling or music	13	Noise outside	2.9
	Loud noise from outside, e.g., bangs and sirens	14	Noise outside	**3.0**
	Buzzing sound of mosquitos	16	Distractions in the bedroom	**3.6**
	Too much light in my bedroom	17	Too light	2.9
	Noise from others at home	21	Noise inside	**3.1**
	Loud sounds from the TV when my parents are watching	25	Noise inside	2.5
	A pet that wakes me when it comes to lie besides me	37	Distractions in the bedroom	2.1
**3. Being unable or reluctant to sleep**				*2.3*
	Not being tired when I go to bed	2	Not tired	**3.9**
	When my parents send me to bed earlier than usual	15	Going to bed too early	2.8
	Being angry because my parents want me to go to sleep	18	Reluctant to go to sleep	2.1
	A bet with my brother/sister about who is able to stay awake the longest	27	Distractions in the bedroom	1.8
	No conversation with my father/mother/brother/sister about what happened that day or what is going to happen the next day before I go to sleep	30	Lacks attention from parents	1.8
	Doing sports late in the evening	32	Evening physical activity	1.5
	Continuing to think about a programme I was watching, but was not allowed to see until the end	34	Negative affective state (frustration)	2.5
	Keep talking with my brother/sister when we are in bed	42	Distractions in the bedroom	2.1
	Wanting to stay up longer	45	Reluctant to go to sleep	2.6
**4. Illness or restless**				*2.7*
	Being ill	3	Illness	2.9
	Not having done much the whole day	4	Inadequate daytime stimulation	2.3
	Not having played outside in the afternoon	5	Inadequate amount of daytime PA	1.9
	Feeling pain	20	Pain	2.9
	Having a blocked nose due to a cold	40	Illness	**3.4**
				
**5.** **Stressful and exciting thoughts and feelings**				2.5
	Being very enthusiastic for the next day	22	Excitement	**3.0**
	Continuing to think about a bothersome event that happened that day	23	Recent stressful event	2.6
	Being bullied at school and thinking about the next day when I am in bed ^b^	36	Being bullied	2.0
	Stress about homework or a test for the next day	39	Upcoming stressful event	**3.3**
	Continuing to think about something exciting that happened that day	41	Excitement	2.1
	Feeling sad ^5^	44	Negative affective state	2.3
**6.Being afraid or uncomfortable in bed**				*2.8*
	Feeling too hot or too cold when I am in bed	6	Not the right temperature	**3.8**
	Watching a scary or moving film in the evening	7	Watching/reading something scary	2.9
	Seeing shadows from outside in my room when I am in bed	9	Scared by something in the bedroom	1.4
	Having scary thoughts when I am in bed	24	Scary thoughts	**3.3**
	Not being able to lie down comfortably	26	Unable to lie down comfortably	**3.8**
	Not sleeping in my favourite sleeping spot in the bunk bed	28	Unfavourable sleeping place	2.1
	Having no comfortable pillow	29	Uncomfortable sleeping materials	2.9
	Reading a moving story before I go to sleep	31	Watching/reading something scary	2.5
	Needing to pee at night	38	Needing to pee	2.8
	Nightmares	43	Nightmares	**3.1**
	Being afraid when I am in bed	46	Being afraid	2.4

^1^ Idea nr. = The number of the generated idea in this group. This corresponds with the numbers on the concept maps in Appendix A. ^2^ Merged into main idea = The overall main idea to which the original idea of this group is assigned. ^3^ Mean = mean importance rating on a 5-point Likert scale based on the question: ‘Think about your sleep, how much does this affect your sleep?’ answered on a 5-point Likert scale from ‘does not affect at all = 1’ to ‘affects a whole lot’ = 5’; ^4^ idea was moved to cluster 1; 5 idea was moved to cluster 5. Bold values indicate ideas rated as important (with a rating ≥3.00); Cursive values indicate the overall mean rating per cluster.

**Table A2:** Cluster compositions of original ideas and mean importance ratings of children in group 2 (*N* = 5), including the assignment of original ideas to main ideas.

Clusters	Original Ideas	Idea nr. ^1^	Merged to Main Idea ^2^	Mean ^3^
**1. Being afraid**				*3.0*
	Watching a scary movie before bedtime	1	Watching/reading something scary	**3.0**
	Nightmares	2	Nightmares	**3.2**
	Stuff in my room that looks scary when I am in bed	21	Scared by something in the bedroom	2.2
	Doing something I find scary at daytime	26	Recent scary event	**3.6**
**2. Stressful and exciting thoughts and feelings, and illness**				*2.9*
	Thinking about something scary when I am in bed	7	Scary thoughts	**3.2**
	In my bed, thinking about bad events that I experienced in the past	11	Recent stressful event	2.6
	Going to do something stressful the next day	12	Upcoming stressful event	**3.0**
	Going to do something exciting the next day	13	Excitement	**3.0**
	Feeling sad when I am in bed	16	Negative affective state	2.8
	Thinking about something sad when I am in bed	17	Negative affective state	2.8
	Being ill	18	Illness	**3.4**
	Feeling pain	19	Pain	**3.0**
	Feeling guilty about something I did that day	27	Negative affective state	**3.0**
	In my bed, thinking about something I experienced that day during sports	28	Recent stressful event	2.4
	Being angry about something that I find unfair	30	Negative affective state	**3.0**
**3. Light in the bedroom**				*2.4*
	A bedroom that is too light	3	Too much light	**3.0**
	A bedroom that is too dark	4	Too dark	1.8
**4. Noise and distractions in the bedroom**				*2.8*
	Loud noise from the neighbours when I am in bed	5	Noise outside	**3.0**
	Loud noise from outside when I am in bed	6	Noise outside	**4.2**
	Scary sounds when I am in bed	8	Noise outside	2.8
	Sleeping together in a bedroom with my brother/sister who makes sounds	9	Distraction in the bedroom	1.8
	A brother or sister that keeps me awake	14	Distraction in the bedroom	**3.0**
	Hearing the sound from the TV when my parents are watching TV and I am in bed	22	Noise inside	2.2
**5. Fear**	Being home alone and afraid that my parents/brother/sister do not come home	15	Scary thoughts	**4.0**
	Being afraid that someone breaks into my home	20	Scary thoughts	**4.0**
	Suddenly not hearing any sounds from my parents in our home when I am in bed	23	Scary thoughts	**3.6**
	Being afraid that my parents are not at home	24	Scary thoughts	**4.0**
	Being afraid that something happens with my parents or someone else in my family	25	Scary thoughts	**4.2**
	Being afraid that someone will laugh at me or say something/will be unkind to me the next day	29	Scary thoughts	2.4

^1^ Idea nr. = The number of the generated idea in this group. This corresponds with the numbers on the concept maps in Appendix A. ^2^ Merged into main idea = The overall main idea to which the original idea of this group is assigned. ^3^ Mean = mean importance rating on a 5-point Likert scale based on the question: ‘Think about your sleep, how much does this affect your sleep?’ answered on a 5-point Likert scale from ‘does not affect at all = 1’ to ‘affects a whole lot’ = 5’. Bold values indicate ideas rated as important (with a rating ≥3.00); Cursive values indicate the overall mean rating per cluster.

**Table A3:** Cluster compositions of original ideas and mean importance ratings of children in group 3A (S1 *N* = 7; S2 *N* = 5), including the assignment of original ideas to main ideas.

Clusters	Original Ideas	Idea nr. ^1^	Merged to Main Idea ^2^	Mean ^3^
**1. Being unable or reluctant to sleep**				*2.5*
	Thinking and having many different thoughts	1	Many thoughts	**3.8**
	watching something on a screen (computer, phone, TV, tablet, laptop) shortly before bedtime	2	Screen use around bedtime	2.0
	Wanting to see something on TV, but not being allowed to see it due to bedtime	3	Negative affective state	2.2
	Messages I receive in the evening via the group chat on my phone	4	Social media use around bedtime	2.0
	When my parents are watching TV and the sound is too loud	6	Noise inside	**3.2**
	My parents making noise in our home ^4^	7	Noise inside	**3.0**
	Wanting to declutter my room before I go to sleep	15	Distraction in the bedroom	1.8
	Not going to bed at the same time every night	16	No consistent sleep schedule	2.6
	Going to bed too early and not being tired enough to fall asleep	17	Going to bed too early	**3.2**
	When I do not do my best to go to sleep	18	Reluctant to go to sleep	2.8
	Not feeling like going to sleep	19	Reluctant to go to sleep	2.5
	Pets making noise in our home	23	Noise inside	1.4
	When an electronic device in my bedroom (e-reader, alarm clock) makes noise	29	Distraction in the bedroom	2.4
	Keep reading for too long	31	Reluctant to go to sleep	2.2
	Doing sports late in the evening	37	Evening physical activity	2.4
	When my brother/sister is allowed to watch something (TV, film) and I am not	40	Fear of missing out	2.0
	Wanting to finish something that I started before going to sleep	41	Reluctant to go to sleep	**3.3**
**2. Uncomfortable in bed**				*3.2*
	Not having the right pillow and therefore not being able to lie down comfortably	5	Uncomfortable sleeping materials	**3.2**
	Not being able to lie down comfortably	30	Unable to lie down comfortably	**3.2**
**3. Illness and discomfort**				*2.9*
	Having difficulty breathing due to hypersensitivity to dust in my bedroom	13	Illness	2.6
	Feeling cold in my bed	14	Not the right temperature	2.4
	Feeling hot in my bed	20	Not the right temperature	**3.6**
	Being sick and not feeling well	21	Illness	**3.3**
	Having a cold and being unable to breath properly	22	Illness	2.8
	Having a dry throat	35	Illness	2.8
**4. Stressful and exciting thoughts and feelings**				*2.9*
	Feeling sad	8	Negative affective state	**3.4**
	Being angry	9	Negative affective state	**3.2**
	Being afraid	10	Negative affective state	2.8
	Being irritated	11	Negative affective state	2.8
	Feeling restless	12	Negative affective state	2.8
	Nightmares and bad dreams	24	Nightmares	**3.2**
	Thinking about bad things ^5^	25	Recent stressful event	**3.0**
	Missing my dad or mum when they are not at home ^5^	26	Negative affective state	**3.2**
	Feeling alone when I am in bed ^5^	27	Negative affective state	2.8
	Hearing weird or scary sounds during the night ^b^	28	Scary sounds	2.6
	Being afraid something scary will happen in my bedroom	32	Scary thoughts	**3.4**
	Looking forward to something fun	33	Excitement	**3.0**
	When I need to pee, but I am too afraid to go to the toilet	34	Scary thoughts	2.4
	Things in my room that suddenly fall down or topples ^b^	36	Scared by something in the bedroom	**3.4**
	When my parents do not pay attention to me because they are busy with my brother or sister ^5^	38	Lacks attention from parents	1.8
	Getting angry because my brother or sister makes noise	39	Negative affective state	2.0

^1^ Idea nr. = The number of the generated idea in this group. This corresponds with the numbers on the concept maps in Appendix A. ^2^ Merged into main idea = The overall main idea to which the original idea of this group is assigned. ^3^ Mean = mean importance rating on a 5-point Likert scale based on the question: ‘Think about your sleep, how much does this affect your sleep?’ answered on a 5-point Likert scale from ‘does not affect at all = 1’ to ‘affects a whole lot’ = 5’; ^4^ idea was moved to cluster 1; ^5^ idea was moved to cluster 4. Bold values indicate ideas rated as important (with a rating ≥3.00); Cursive values indicate the overall mean rating per cluster. S1 = Session 1, brainstorm; S2 = Session 2, sort and rate.

**Table A4:** Cluster compositions of original ideas and mean importance ratings of children in group 3B (S1 *N* = 7; S2 *N* = 8), including the assignment of original ideas to main ideas.

Clusters	Original Ideas	Idea nr. ^1^	Merged to Main Idea ^2^	Mean ^3^
**1. Stressful and exciting thoughts and feelings**				*3.1*
	Looking forward to something that will happen the next day	1	Excitement	**3.4**
	Thinking and having many thoughts	2	Many thoughts	**4.3**
	Being nervous for something that is going to happen	3	Upcoming stressful event	2.9
	Thinking about which secondary school I will go to	4	Upcoming stressful event	2.5
	Thinking about my parents, who were in a fight	8	Stressful family situation	2.4
	Thinking about my share in a fight at school and how I could do things better next time	9	Recent stressful event	2.5
	Being enthusiastic about something that happened that day	11	Excitement	2.9
	Thinking about not being able to sleep well	23	Upcoming stressful event	2.8
	Thinking about the divorce of my parents	24	Stressful family situation	2.0
	Being bullied and thinking about this when I am in bed	28	Recent stressful event	2.5
	When something stressful is going to happen	29	Upcoming stressful event	**3.6**
**2. Noise and distractions in the bedroom**				*2.3*
	When someone in our home is watching TV and the sound is too loud	13	Noise inside	2.4
	Noise from a brother or sister with whom I share the same room	20	Distractions in the bedroom	1.9
	Noise from the street	31	Noise outside	2.4
	Noise inside our house	32	Noise inside	2.5
	When my phone beeps at night due to games or messages	42	Distractions in the bedroom	1.5
	The buzzing and stinging of mosquitos	45	Distractions in the bedroom	**3.3**
	Not feeling comfortable because of people screaming outside	46	Feeling unsafe	2.3
	Ticking of the clock in my bedroom	48	Distractions in the bedroom	2.1
**3. Illness and discomfort**	Illness and not feeling well	7	Illness	**3.5**
	Not being able to lie down comfortably in my bed	10	Unable to lie down comfortably	2.4
	Having had too much to eat	14	Inadequate dietary behaviour	2.3
	Urgently needing to pee	22	Needing to pee	2.6
	Feeling too cold in bed	25	Not the right temperature	2.9
	Feeling too hot in bed	26	Not the right temperature	2.6
	Feeling pain	39	Pain	2.5
	Being thirsty	41	Inadequate dietary behaviour	2.3
**4. Emotions**				*2.6*
	Nightmares	30	Nightmares	2.8
	Being afraid	36	Being afraid	2.6
	Being very happy	37	Excitement	2.9
	Being angry	38	Negative affective state	2.4
	Being afraid that someone comes into my home	44	Being afraid	2.1
**5. Unable or reluctant to sleep**				*2.2*
	Having had too little exercise during the day and therefore too much energy in the evening	5	Inadequate amount of daytime physical activity	2.5
	When my pet comes into my bedroom and distracts me when I am in bed	6	Distractions in the bedroom	1.5
	Watching a scary movie and continuing to think about it ^4^	12	Watching/reading something scary	2.6
	Having had too little too eat and feeling hungry ^4^	15	Inadequate dietary behaviour	2.6
	Being too tired because I did a lot	17	Excessive daytime stimulation	1.9
	Not feeling like going to sleep ^4^	18	Reluctant to go to sleep	2.4
	Going to bed too early and not being tired	19	Going to bed too early	**3.6**
	Going to bed too late and not feeling sleepy any more	21	No consistent sleep schedule	2.3
	Watching TV right before going to sleep	33	Screen use around bedtime	2.0
	Playing computer games right before going to sleep	34	Playing active games before bedtime	2.0
	Using a screen with blue light (TV, computer, laptop, tablet) right before going to sleep	35	Screen use around bedtime	2.3
	Being really tired and getting activated when I brush my teeth	40	No consistent sleep schedule	1.8
	Scary sounds ^4^	43	Scary sounds	2.4
	Too much light in my bedroom	47	Too light	2.4
	Lying in my mother’s bed and looking out the window ^4^	49	Distractions in the bedroom	1.5
**6. General negative state of mind**				*2.5*
	When I feel gloomy and not in the mood to do anything	16	Negative affective state	2.6
	Feeling insecure about myself	27	Negative affective state	2.4

^1^ Idea nr. = The number of the generated idea in this group. This corresponds with the numbers on the concept maps in Appendix A. ^2^ Merged into main idea = The overall main idea to which the original idea of this group is assigned. ^3^ Mean = mean importance rating on a 5-point Likert scale based on the question: ‘Think about your sleep, how much does this affect your sleep?’ answered on a 5-point Likert scale from ‘does not affect at all = 1’ to ‘affects a whole lot’ = 5’; ^4^ idea was moved to cluster 5. Bold values indicate ideas rated as important (with a rating ≥3.00); Cursive values indicate the overall mean rating per cluster. S1 = Session 1, brainstorm; S2 = Session 2, sort and rate.

**Table A5:** Cluster compositions of original ideas and mean importance ratings of children in group 4A (*N* = 7), including the assignment of original ideas to main ideas.

Clusters	Original Ideas	Idea nr. ^1^	Merged to Main Idea ^2^	Mean ^3^
**1. Stressful thoughts and feelings**				*2.2*
	When I had a fight with classmates and the fight has not been resolved yet	1	Recent stressful event	2.0
	Being bullied and thinking about it when I am in bed	5	Recent stressful event	2.6
	Being in a fight with my father, mother, brother or sister and thinking about it when I am in bed	22	Recent stressful event	**3.1**
	Thinking about being kicked out of class at school by my teacher	27	Recent stressful event	1.7
	Being hurt by a classmate	32	Recent stressful event	2.0
	Not having had a nice day	43	Recent stressful event	2.4
	Receiving unpleasant messages on social media	45	Recent stressful event	1.6
**2. Noise, distractions, and stressful family situation**				*2.9*
	When my parents make too much noise in our home	3	Noise inside	**3.9**
	Screaming noise from the neighbours	13	Noise outside	2.9
	Noise from outside	16	Noise outside	**3.0**
	When my parents are fighting and screaming	17	Stressful family situation	**3.4**
	The sound of whizzing in my ears when it is very quiet ^4^	21	Distractions in the bedroom	2.1
	Feeling sad	29	Negative affective state	2.9
	When I hear my father and mother doing dirty things	35	Noise inside	2.7
	A brother or sister that keeps me awake	41	Distractions in the bedroom	2.9
	The sound of the TV is too loud when I am in bed ^5^	42	Noise inside	2.9
**3. Unable or reluctant to sleep**				*2.7*
	Being nervous for the next day, because something fun is going to happen	4	Excitement	**3.3**
	When I go to bed too early and I am not tired yet	7	Going to bed too early	**3.3**
	Doing sports until late	8	Evening physical activity	1.9
	Going to bed very late	10	No consistent sleep schedule	1.9
	Having slept during the day and therefore not feeling tired at night	18	Daytime nap	2.7
	Feeling restless	20	Negative affective state	**3.1**
	Feeling too hot	24	Not the right temperature	**3.6**
	Still feeling like playing ^6^	26	Reluctant to go to sleep	**3.0**
	Still having too much energy	37	Not tired	**3.1**
	Using my phone right before going to sleep	44	Screen use around bedtime	2.1
	Secretly using the tablet after bedtime	46	Screen use around bedtime	1.7
**4. Stressed for the upcoming day**				*3.5*
	When there are important and stressful events planned	31	Upcoming stressful event	**3.0**
	Going to do something stressful the next day	33	Upcoming stressful event	**4.2**
	Needing to do something I do not want to do the next day	34	Upcoming stressful event	**3.3**
**5. Discomfort**				*3.1*
	Late dinner	9	Inadequate dietary behaviour	2.0
	Needing to pee when I am already in bed ^7^	11	Needing to pee	2.7
	Having had too much to eat	14	Inadequate dietary behaviour	2.7
	Having had too many different things to eat	15	Inadequate dietary behaviour	2.6
	Not feeling well	19	Illness	**3.9**
	Feeling hungry	23	Inadequate dietary behaviour	**3.6**
	Muscle strain	36	Pain	**3.7**
	Feeling pain	39	Pain	**3.4**
**6. Being afraid**				*2.7*
	Being afraid because of scary things that my father or mother told me	2	Being afraid	2.6
	In bed, thinking about something scary that I experienced	6	Recent scary event	**3.7**
	Having seen something scary on TV before going to bed	12	Watching/reading something scary	**3.0**
	Having seen something scary at daytime	25	Recent scary event	2.4
	Having seen a scary movie	28	Watching/reading something scary	**3.1**
	Not feeling safe	30	Feeling unsafe	2.2
	Having a nightmare	38	Nightmares	**3.0**
	Being afraid in the dark and preferring a light in my room	40	Being afraid	1.6

^1^ Idea nr. = The number of the generated idea in this group. This corresponds with the numbers on the concept maps in Appendix A. ^2^ Merged into main idea = The overall main idea to which the original idea of this group is assigned. ^3^ Mean = mean importance rating on a 5-point Likert scale based on the question: ‘Think about your sleep, how much does this affect your sleep?’ answered on a 5-point Likert scale from ‘does not affect at all = 1’ to ‘affects a whole lot’ = 5’; ^4^ idea was moved to cluster 2; ^5^ idea was moved from cluster 1 to cluster 2;.^6^ idea was moved to from cluster 4 to cluster 3; ^7^ idea was moved to from cluster 2 to cluster 5. Bold values indicate ideas rated as important (with a rating ≥3.00); Cursive values indicate the overall mean rating per cluster.

**Table A6:** Cluster compositions of original ideas and mean importance ratings of children in group 4B (S1 *N* = 7; S2 *N* = 9), including the assignment of original ideas to main ideas.

Clusters	Original Ideas	Idea nr. ^1^	Merged to Main Idea ^2^	Mean ^3^
**1. Unable or reluctant to sleep**				*2.6*
	Having a jetlag after a holiday due to the time difference	1	No consistent sleep schedule	2.2
	When my room is too hot or too cold	5	Not the right temperature	2.9
	Feeling hungry	7	Inadequate dietary behaviour	**3.2**
	Being too busy for too long, because I want to do so many things in one day	9	Excessive daytime stimulation	2.2
	Needing to pee when I am already sleeping	10	Needing to pee	2.4
	Having a cold and a blocked nose	11	Illness	**3.3**
	Being ill	12	Illness	**3.0**
	Sleeping on an uncomfortable mattress	19	Uncomfortable bed	2.9
	Not wanting to go to sleep	22	Reluctant to go to sleep	**3.6**
	Feeling thirsty	26	Inadequate dietary behaviour	**3.2**
	Sleeping at daytime ^4^	27	Daytime nap	2.0
	Mosquitos and their bites that bother me	28	Distraction in the bedroom	**4.2**
	Feeling pain	31	Pain	2.2
	Going to sleep with wet hair	33	Unable to lie down comfortably	**3.1**
	Too much light in my bedroom	34	Too light	2.9
	When I do not feel comfortable with the person I am going to stay with for the night	35	Feeling unsafe	2.4
	Having had too much to eat	36	Inadequate dietary behaviour	2.0
	Having had too much sugar	37	Inadequate dietary behaviour	1.8
	Wearing uncomfortable pyjamas	39	Uncomfortable sleeping materials	2.7
	Feeling homesick when I sleep somewhere else	40	Feeling unsafe	1.8
	Unable to lay down comfortably due to my hair	41	Unable to lie down comfortably	**3.3**
	Seeing toys in my bedroom and feeling like playing	45	Distraction in the bedroom	1.4
	Seeing food next to my bed and feeling like eating	46	Distraction in the bedroom	1.9
	Smelling strange odours in my bedroom	54	Distraction in the bedroom	1.7
	Light from outside that shines into my bedroom	55	Too light	2.7
	Having a falling sensation when I am sleeping	57	Nightmares	2.7
**2. Noise and distractions in the bedroom**				*2.6*
	When the sound of the TV in our home is too loud	15	Noise inside	2.4
	Pets that wake me up	30	Distraction in the bedroom	2.0
	Noise from the neighbours or outside	43	Noise outside	**3.1**
	A brother or sister who makes noise in our home	47	Noise inside	2.3
	When we have visitors in our home	48	Noise inside	**3.0**
**3. Stressful and exciting thoughts and feelings and stressful family situation**				*2.7*
	When the next day is a special day	2	Excitement	**3.1**
	Being in a fight with my parents and the fight has not been resolved yet	6	Recent stressful event	**3.3**
	Having too much on my mind	8	Many thoughts	2.8
	Being enthusiastic about something that is going to happen	13	Excitement	**3.2**
	Not looking forward to the next day, because something bad can happen	16	Upcoming stressful event	2.6
	Thinking about my parents who are divorced	17	Stressful family situation	1.0
	Thinking about someone I love and is not amongst us anymore ^5^	20	Negative affective state	**3.67**
	When I had a very nice dream and I keep thinking about it	21	Excitement	2.1
	When something very bad happened that day	23	Recent stressful event	**3.3**
	Needing to go to the doctor the next day	24	Upcoming stressful event	2.1
	Being afraid that someone enters my room	29	Being afraid	**3.2**
	When I did something bad that can get me into trouble	38	Recent stressful event	2.8
	Hearing my parents talk about difficult stuff when I am in bed	42	Stressful family situation	1.8
	Thinking about something difficult that happened at school	44	Recent stressful event	2.9
	Hearing my parents fight when I am in bed	49	Stressful family situation	2.3
	Having a test or classroom presentation the next day	52	Upcoming stressful event	**3.1**
	When I did not finish my homework for the next day	53	Upcoming stressful event	2.8
	Desire to take revenge when someone did something bad to me	56	Negative affective state	2.9
	When I did a test and it did not go well	58	Recent stressful event	2.4
**4. Agitating activities before bedtime and being afraid**				*2.7*
	A nightmare	3	Nightmares	**3.8**
	Using my phone right before I go to sleep	4	Screen use around bedtime	1.9
	When I heard a scary story	14	Recent scary event	**3.3**
	Having watched a scary movie	18	Watching/reading something scary	**3.7**
	When I see scary shadows in my bedroom	25	Scared by something in the bedroom	**3.3**
	Reading a scary book	32	Watching/reading something scary	1.8
	Using my phone or tablet in my bed	50	Screen use around bedtime	2.3
	Having watched TV before I go to bed	51	Screen use around bedtime	1.4

^1^ Idea nr. = The number of the generated idea in this group. This corresponds with the numbers on the concept maps in Appendix A. ^2^ Merged into main idea = The overall main idea to which the original idea of this group is assigned. ^3^ Mean = mean importance rating on a 5-point Likert scale based on the question: ‘‘Think about your sleep, how much does this affect your sleep?’ answered on a 5-point Likert scale from ‘does not affect at all = 1’ to ‘affects a whole lot’ = 5’; ^4^ idea was moved from cluster 2 to cluster 1; ^5^ idea was moved from cluster 2 to cluster 3. Bold values indicate ideas rated as important (with a rating ≥3.00); Cursive values indicate the overall mean rating per cluster. S1 = Session 1, brainstorm; S2 = Session 2, sort and rate.

**Table A7:** Cluster compositions of original ideas and mean importance ratings of parents in group 1 (S1 *N* = 4; S2 *N* = 7), including the assignment of original ideas to main ideas.

Clusters	Original Ideas	Idea nr. ^1^	Merged to Main Idea ^2^	Mean ^3^
**1. Energy, diet, and physical activity**				3.3
	Being home all day and therefore not being tired	1	Inadequate daytime stimulation	**3.6**
	Being too tired when going to bed	2	Too tired	**3.3**
	Not having played outside at daytime	5	Inadequate time outside at daytime	**3.0**
	Feeling hungry during the night	10	Inadequate amount of food	2.6
	Eating too much candy and/or drinks with a lot of sugar	11	Unhealthy diet	**3.4**
	Eating too much before bedtime	12	Excessive amount of food close to bedtime	**3.3**
	Having too much energy from his/herself	20	Too energetic	**3.9**
**2. Stressful and exciting thoughts and feelings, physical well-being, routines and screen time behaviour**				*3.4*
	A stressful event coming up for the child the next day	3	Upcoming stressful event	**4.0**
	Exciting activities planned the next day	4	Excitement	**3.6**
	Long nights during the days with more hours of darkness	6	Seasonal changes	2.9
	The transition from long days to short days or the other way around	7	Seasonal changes	**3.0**
	Not willing to go to sleep	13	Reluctant to go to sleep	**3.0**
	Playing with the tablet or phone before bedtime	14	Screen use before bedtime	**3.4**
	Being not allowed to play with their phone before bedtime, when the child normally always does this	15	Deviate from bedtime routine	2.6
	In bed thinking about what happened that day, when a child did not talk about their day	26	Worrying	**3.6**
	Being ill	28	Illness	**4.1**
	Sleep walking	29	Sleep walking	**3.0**
	No consistent sleep times / sleep schedule	30	No consistent sleep schedule	**3.9**
	Having watched a scary movie	32	Watching something scary	**3.4**
**3. Stressful family or school situation**				*3.7*
	A stressful family situation due to a fight between parents	19	Parental relationship problems	**3.4**
	Parental feeling of anger or stress which is transferred to the child	24	Parental stress	**3.9**
	Having had a fight at school and still and still worrying about that	25	Recent stressful event	**3.9**
	Feeling insecure about his/herself	27	Insecurity about themselves	**4.0**
	Being bullied at school	31	Being bullied	**3.4**
**4. Sleep environment**				
	A bedroom with too much light	8	Too much light	2.7
	A bedroom that is too dark	9	Too dark	2.9
	Having to sleep alone, while the child actually wants to sleep with his/her parents	16	Reluctant to sleep alone	**3.3**
	Being distracted due to toys in the bedroom	17	Distractions in the bedroom	2.4
	Sleeping in one room with a brother/sister who keeps the child awake	18	Distractions in the bedroom	2.7
	A less tidy or clean house than usual	21	Distractions in the bedroom	**3.0**
	A less tidy and messy bedroom	22	Distractions in the bedroom	2.9
	A bedroom which is too hot or too cold	23	Not the right temperature	**3.1**

^1^ Idea nr. = The number of the generated idea in this group. This corresponds with the numbers on the concept maps in Appendix A. ^2^ Merged into main idea = The overall main idea to which the original idea of this group is assigned. ^3^ Mean = mean importance rating on a 5-point Likert scale based on the question: ‘Think about the sleep of a child in the age of 4-12 years, how much does this affect their sleep?’ answered on a 5-point Likert scale from ‘does not affect at all = 1’ to ‘affects a whole lot = 5’. Bold values indicate ideas rated as important (with a rating ≥3.00); Cursive values indicate the overall mean rating per cluster. S1 = Session 1, brainstorm; S2 = Session 2, sort and rate.

**Table A8:** Cluster compositions of original ideas and mean importance ratings of parents in group 2 (S1 *N* = 8; S2 *N* = 9), including the assignment of original ideas to main ideas.

Clusters	Original Ideas	Idea nr. ^1^	Merged to Main Idea ^2^	Mean ^3^
**1. Diet, physical activity, and screen behaviour**				*3.5*
	Having had too much sugar	1	Unhealthy diet	**3.7**
	Having watched a scary movie	8	Watching something scary	**4.0**
	Inadequate amount of physical activity during the day and therefore not being tired	9	Inadequate amount of daytime physical activity	**3.6**
	Using the TV before bedtime	13	Screen use before bedtime	**3.1**
	Using a phone before bedtime	14	Screen use before bedtime	**3.2**
	Using the computer before bedtime	15	Screen use before bedtime	**3.3**
	Using a tablet before bedtime	16	Screen use before bedtime	**3.3**
	Late dinner	21	Excessive amount of food close to bedtime	**3.4**
	Having had too much to eat right before going to bed	22	Excessive amount of food close to bedtime	**3.6**
	Unhealthy diet during the day	23	Unhealthy diet	**3.6**
	Before bedtime, playing with toys with a lot of light and noise	42	Play with activating toys before bedtime	**3.1**
**2. Noise from the neighbours**				*3.6*
	Screaming kids from the neighbours	39	Noise outside	**3.7**
	Fighting neighbours	40	Noise outside	**3.8**
	Noise from the neighbours’ pets	41	Noise outside	**3.2**
**3. Mental distress, physical well-being, and stressful family situation**				
	Absence of the mother or father when the child needs attention	2	Parental absence when child needs attention	**4.0**
	A stressful event coming up for the child the next day	3	Upcoming stressful event	**3.9**
	An important event coming up for the child the next day	4	Upcoming stressful event	**3.8**
	Being ill	5	Illness	**4.22**
	Not being able to breathe well due to a cold	6	Illness	**4.2**
	Having had a fight at school and still worrying about that	10	Recent stressful event	**3.2**
	Worrying about something and not being able to share this	20	Worrying	**3.3**
	Home visits from family or friends	27	Fear of missing out	**3.9**
	Something fun coming up the next day	43	Excitement	**3.6**
	Financial problems at home, causing that the parent is not able to buy everything for the child	44	Financial family problems	2.6
	Having nothing to look forward to the next day	46	Worrying	2.6
	Having done something naughty at daytime	47	Recent stressful event	2.6
	Being bullied	48	Being bullied	**3.8**
	Parents going through a divorce	49	Parental relationship problems	**3.8**
	Parents who are fighting	50	Parental relationship problems	**4.1**
	Being jealous of a friend or classmate	51	Worrying	2.9
	Parents who do not live together any more	52	Parental relationship problems	**3.4**
	Being afraid when lying in bed	53	Being afraid	**3.6**
**4. Routines and social environment**				*3.5*
	Having a longer night sleep than usual the night before, because the child woke up later	7	No consistent sleep schedule	**4.00**
	Irregular sleep times during weekends	11	No consistent sleep schedule	**3.1**
	Not indicating clearly when the child needs to go to bed	17	Indistinctness about bedtime	**3.2**
	The change of the time due to summer and wintertime	24	No consistent sleep schedule	**3.7**
	Holidays or having a free day the next day	32	No consistent sleep schedule	**3.2**
	A brother or sister that is allowed to go to bed later	33	Social norm for bedtime among siblings	**4.2**
	Classmates that have a later bedtime	34	Social norm for bedtime among classmates	2.8
**5. Sleep environment and bedtime routine**				*3.1*
	Not reading a story to the child, when this normally does happen	12	Deviate from usual bedtime routine	2.7
	Not having a bedtime routine	18	No bedtime routine	**3.3**
	Having napped in the afternoon	19	Daytime nap	**3.0**
	When it is still light outside when they need to go to bed	25	Seasonal changes	**4.0**
	Sleeping at a different place than his/her own bed	26	Different sleep environment	**3.0**
	Not having a comfortable bed and therefore not being able to lie down comfortably	28	Uncomfortable place to sleep	**3.0**
	A bedroom that is too hot or too cold	29	Not the right temperature	**3.4**
	Uncomfortable sleeping attributes, such as pillows, pyjamas, underwear	30	Uncomfortable sleeping material	**3.1**
	The absence of the favourite stuffed animal or sleeping cloth	31	Absence of favourite sleep accessory	2.7
	Too much light in the bedroom	35	Too much light	**3.4**
	A bedroom that is too dark	36	Too dark	2.9
	Busy decor in the bedroom	37	Distractions in the bedroom	**3.0**
	No fresh air in the bedroom	38	No fresh air	**3.0**

^1^ Idea nr. = The number of the generated idea in this group. This corresponds with the numbers on the concept maps in Appendix A. ^2^ Merged into main idea = The overall main idea to which the original idea of this group is assigned. ^3^ Mean = mean importance rating on a 5-point Likert scale based on the question: ‘Think about the sleep of a child in the age of 4-12 years, how much does this affect their sleep?’ answered on a 5-point Likert scale from ‘does not affect at all = 1’ to ‘affects a whole lot = 5’. Bold values indicate ideas rated as important (with a rating ≥3.00); Cursive values indicate the overall mean rating per cluster. S1 = Session 1, brainstorm; S2 = Session 2, sort and rate.

**Table A9:** Cluster compositions of original ideas and mean importance ratings of parents in group 3 (S1 *N* = 8; S2 *N* = 9), including the assignment of original ideas to main ideas.

Clusters	Original Ideas	Idea nr. ^1^	Merged to Main Idea ^2^	Mean ^3^
**1. Mental distress and active brain**				*3.4*
	Having many thoughts	1	Many thoughts	**4.2**
	Having all kinds of questions before bedtime	2	Many thoughts	**3.0**
	Wanting to solve world problems before bedtime	3	Many thoughts	**3.9**
	Continuing to think about something that is about to happen the next day	4	Upcoming stressful event	**3.6**
	Continuing to think about something that happened that day	5	Recent stressful event	**3.6**
	Having many ideas and wanting to realize these before going to sleep	6	Many thoughts	**3.8**
	Indistinctness about what is going to happen the next day	7	Many thoughts	**3.4**
	Being too tired (overtired) before bedtime and getting hyperactive due to that	10	Too tired	**3.6**
	When the child has planned to do something that day and tis still needs to happen before going to bed	18	Many thoughts	**3.1**
	Not willing to end the day, because it was such a fun day	24	Many thoughts	2.6
	Curiosity about how sleep works, wondering when and if you will wake up	28	Many thoughts	2.0
	Not being able to let go of feelings of injustice before bedtime	42	Many thoughts	**3.7**
**2. Family- and social environment**				*3.1*
	When the parent rushes to take the child to bed	8	Parental stress	**3.1**
	When the parent deviates from the usual bedtime routine	9	Deviate from usual bedtime routine	**3.4**
	Going to bed too late	19	No consistent sleep schedule	**3.3**
	When the sleep schedule gets disturbed	20	No consistent sleep schedule	**3.4**
	Older brothers or sisters that are allowed to stay up longer	21	Social norm for bedtime among siblings	2.7
	Other children in their class that are allowed to stay up longer	22	Social norm for bedtime among classmates	2.3
	When there are visitors at home	23	Fear of missing out	**3.1**
	Wanting to stay with their parent and not wanting to be alone	33	Reluctant to sleep alone	**3.2**
**3. Sleep environment**				*2.8*
	Noise from the street	11	Noise outside	2.9
	Absence of the favourite stuffed animal or sleeping cloth	29	Absence of favourite sleep accessory	**3.1**
	A dark bedroom	32	Too dark	2.9
	When it is too quiet at home	38	Too quiet	2.1
	Noises from in or outside the house that are different than usual	39	Noise outside	**3.0**
**4. Stressful family situation, worries and fear**				*3.9*
	Parents who are in a fight	12	Parental relationship problems	**4.7**
	Relationship problems between parents	13	Parental relationship problems	**4.3**
	Worrying about things that are going on at home	14	Worrying	**3.8**
	When something is going on with (one of) their friends	15	Worrying	**3.6**
	Not feeling safe at home, in the classroom or outside on the streets	17	Feeling unsafe	**4.1**
	Being afraid that someone comes into their home or something happens with their home	27	Being afraid	**3.1**
	Having watched a scary movie	30	Watching something scary	**3.9**
	Having watched the news	31	Watching something scary	**3.3**
**5. physical- and mental well-being**				*3.7*
	When other children were unkind and still worrying about this	16	Being bullied	**4.2**
	Feeling pain	25	Pain	**4.2**
	Being ill	26	Illness	**4.2**
	Having had inadequate stimulation during the day	34	Inadequate daytime stimulation	**3.1**
	Having had excessive stimulation during the day	35	Excessive daytime stimulation	**3.4**
	Dreams that keep children awake or wake them and cause restless sleep	36	Unpleasant dreams	**3.3**
	When something happens in the light sleep phase, waking the child and giving opportunity to start thinking	37	Many thoughts	**3.0**
**6. Screen behaviour before bedtime**				*4.1*
	Activating computer games that cause stress before bedtime ^4^	40	Play with activating toys before bedtime	**4.4**
	The light of screens (tablet, computer) right before going to sleep ^4^	41	Screen use before bedtime	**3.7**

^1^ Idea nr. = The number of the generated idea in this group. This corresponds with the numbers on the concept maps in Appendix A. ^2^ Merged into main idea = The overall main idea to which the original idea of this group is assigned. ^3^ Mean = mean importance rating on a 5-point Likert scale based on the question: ‘Think about the sleep of a child in the age of 4-12 years, how much does this affect their sleep?’ answered on a 5-point Likert scale from ‘does not affect at all = 1’ to ‘affects a whole lot = 5’; ^4^ a new cluster was created with this idea. Bold values indicate ideas rated as important (with a rating ≥3.00); Cursive values indicate the overall mean rating per cluster. S1 = Session 1, brainstorm; S2 = Session 2, sort and rate.

**Table A10:** Cluster compositions of original ideas and mean importance ratings of parents in group 4 (S1 *N* = 7; S2 *N* = 6), including the assignment of original ideas to main ideas.

Clusters	Original Ideas	Idea nr. ^1^	Merged to Main Idea ^2^	Mean ^3^
**1. Sleep environment**				*3.1*
	Too much noise within the home	7	Noise inside	**3.3**
	Too much noise from outside	8	Noise outside	**3.5**
	A bedroom that is too light due to incoming light	9	Too much light	**3.0**
	When there is still daylight when the child needs to go to sleep	10	Seasonal changes	**3.2**
	Too much distraction due to too many stuffed animals or other toys in bed	19	Distractions in the bedroom	2.3
	Pets in the bedroom that cause distractions	21	Distractions in the bedroom	**3.2**
	Hearing sounds that are different than usual when the child lies in bed	29	Noise inside	2.7
	Not able to lay down comfortably due to a hard surface	30	Uncomfortable place to sleep	**3.0**
	Feeling too hot when the child lies in bed	44	Not the right temperature	**3.0**
	Feeling too cold when the child lies in bed	45	Not the right temperature	**3.2**
	Mosquitos in the bedroom	46	Distractions in the bedroom	**3.7**
	Shared bedroom with brothers or sisters	52	Distractions in the bedroom	2.7
**2. Mental distress, stressful family situation, worries, and fear**				*4.0*
	Having too much on his/her mind due to many thoughts	3	Many thoughts	**4.2**
	Continuing to analyse past events when the child lies in bed	4	Recent stressful event	**4.5**
	Being angry at older brothers and sisters that are allowed to stay up longer	11	Social norm for bedtime among siblings	2.8
	Excitement, happy feelings for something that is going to happen the next day	12	Excitement	**3.2**
	Being nervous for something that is going to happen the next day	13	Upcoming stressful event	**3.5**
	Being bullied	15	Being bullied	**4.7**
	Having nightmares	31	Unpleasant dreams	**4.5**
	Feeling left out because the child is not allowed to stay up	32	Fear of missing out	**3.3**
	Not willing to miss something and therefore not willing to go to sleep	33	Fear of missing out	**3.0**
	Fear for own ideas and fantasies	34	Being afraid	**4.5**
	Having watched a scary movie and after that being afraid when lying in bed	35	Watching something scary	**3.8**
	When the child feels unsafe	36	Feeling unsafe	**4.7**
	Parental stress (rushing) that is transferred to the child	48	Parental stress	**4.3**
	Negative tension between parents	49	Parental relationship problems	**4.3**
	Negative tension or disagreement within the family	50	Parental relationship problems	**4.5**
	Missing the feeling of security when the child needs to sleep alone and was used to sleep with a sibling	53	Reluctant to sleep alone	**3.0**
	Being afraid in the dark	55	Being afraid	**4.5**
	Worrying about things that did not go well during the day and not being able to let it go	56	Recent stressful event	**4.0**
**3. Family environment and sleep habits**				*3.4*
	When a parent is (not able to be) present at the moment the child needs it	24	Parental absence when child needs attention	2.8
	Waiting for a parent that still needs to come home	25	Parental absence when child needs attention	**3.0**
	A different environment, not sleeping in their own bed	28	Different sleep environment	**3.0**
	Having wet the bed	43	Sleep problem	**4.0**
	Absence of the favourite stuffed animal or sleeping cloth	54	Absence of favourite sleep accessory	**4.2**
**4. Energy, diet, physical activity, and routines**				*3.3*
	A busy day with excessive stimulation due to too many activities	1	Excessive daytime stimulation	**4.0**
	A boring day with inadequate stimulation due to lack of activities	2	Inadequate daytime stimulation	**3.2**
	Not being tired when going to bed	5	Too energetic	**3.8**
	Being too tired when going to bed	6	Too tired	2.8
	Did not have physical activity (sports, playing outside) after school and were therefore not able to use their energy	14	Inadequate amount of daytime physical activity	**3.2**
	Feeling hungry	16	Inadequate amount of food	**3.2**
	Feeling thirsty	17	Did not drink enough	**3.2**
	When they are not feeling like going to sleep	18	Reluctant to go to sleep	**3.3**
	A bedtime that is too early for the child’s circadian rhythm	37	Too early bedtime	**3.8**
	Having napped at daytime	39	Daytime nap	**3.5**
	Having had a peak of energy before going to sleep and therefore being too energetic at bedtime	40	Too energetic	**3.7**
	Having had something to eat with a lot of sugar before bedtime	41	Unhealthy diet	2.5
	Having had too much to drink before going to sleep and therefore needing to go to the toilet often	42	Drinking too much before bedtime	**3.2**
	Having had dinner shortly before bedtime	47	Excessive amount of food close to bedtime	2.7
	Having a jetlag ^4^	51	No consistent sleep schedule	2.8
	Spending too little time outside in the fresh air	57	Inadequate time outside at daytime	**3.3**
	Lack of water during the day	58	Did not drink enough	**3.0**
**5. Screen behaviour and bedtime routine**				*3.4*
	When the phone is placed by the bed and causes distraction due to the sound of incoming messages	20	Distractions in the bedroom	**4.0**
	Using the computer or other screen (phone, tablet, game computer, TV) right before going to sleep	22	Screen use before bedtime	**3.3**
	Using screens (phone, tablet, game computer, TV) a lot during the day	23	Excessive daytime screen use	2.7
	Disturbance of the bedtime routine, activities are different than usual	26	Deviate from usual bedtime routine	**3.5**
	Having no bedtime routine	27	No bedtime routine	**3.7**
**6. Physical well-being**				*3.5*
	Being ill ^5^	38	Illness	**3.5**

^1^ Idea nr. = The number of the generated idea in this group. This corresponds with the numbers on the concept maps in Appendix A. ^2^ Merged into main idea = The overall main idea to which the original idea of this group is assigned. ^3^ Mean = mean importance rating on a 5-point Likert scale based on the question: ‘Think about the sleep of a child in the age of 4-12 years, how much does this affect their sleep?’ answered on a 5-point Likert scale from ‘does not affect at all = 1’ to ‘affects a whole lot = 5’; ^4^ this idea was moved from cluster 5 to cluster 4; ^5^ this idea was subtracted from cluster 5. Bold values indicate ideas rated as important (with a rating ≥3.00); Cursive values indicate the overall mean rating per cluster. S1 = Session 1, brainstorm; S2 = Session 2, sort and rate.
